# *Dstyk* mutation leads to congenital scoliosis-like vertebral malformations in zebrafish via dysregulated mTORC1/TFEB pathway

**DOI:** 10.1038/s41467-019-14169-z

**Published:** 2020-01-24

**Authors:** Xianding Sun, Yang Zhou, Ruobin Zhang, Zuqiang Wang, Meng Xu, Dali Zhang, Junlan Huang, Fengtao Luo, Fangfang Li, Zhenhong Ni, Siru Zhou, Hangang Chen, Shuai Chen, Liang Chen, Xiaolan Du, Bo Chen, Haiyang Huang, Peng Liu, Liangjun Yin, Juhui Qiu, Di Chen, Chuxia Deng, Yangli Xie, Lingfei Luo, Lin Chen

**Affiliations:** 1 Department of Wound Repair and Rehabilitation, State Key Laboratory of Trauma, Burns and Combined Injury, Trauma Center, Research Institute of Surgery, Daping Hospital, Army Medical University, Chongqing, 400042 China; 2 Key Laboratory of Freshwater Fish Reproduction and Development, Ministry of Education, Laboratory of Molecular Developmental Biology, School of Life Sciences, Southwest University, Beibei, Chongqing 400715 China; 3 Department of Orthopedic Surgery, The Second Affiliated Hospital, Chongqing Medical University, Chongqing, 400010 China; 4 College of Bioengineering, Chongqing University, Chongqing, 400044 China; 5 Department of Orthopedic Surgery, Rush University Medical Center, Chicago, IL 60612 USA; 6 Genetics of Development and Disease Branch, National Institute of Diabetes, Digestive and Kidney Diseases, National Institutes of Health, Bethesda, MD 20892 USA; 7 Faculty of Health Sciences, University of Macau, Macau SAR, China

**Keywords:** Lysosomes, Vacuole, Bone development, Disease model, Development

## Abstract

Congenital scoliosis (CS) is a complex genetic disorder characterized by vertebral malformations. The precise etiology of CS is not fully defined. Here, we identify that mutation in *dual serine/threonine and tyrosine protein kinase* (*dstyk*) lead to CS-like vertebral malformations in zebrafish. We demonstrate that the scoliosis in *dstyk* mutants is related to the wavy and malformed notochord sheath formation and abnormal axial skeleton segmentation due to dysregulated biogenesis of notochord vacuoles and notochord function. Further studies show that DSTYK is located in late endosomal/lysosomal compartments and is involved in the lysosome biogenesis in mammalian cells. *Dstyk* knockdown inhibits notochord vacuole and lysosome biogenesis through mTORC1-dependent repression of TFEB nuclear translocation. Inhibition of mTORC1 activity can rescue the defect in notochord vacuole biogenesis and scoliosis in *dstyk* mutants. Together, our findings reveal a key role of DSTYK in notochord vacuole biogenesis, notochord morphogenesis and spine development through mTORC1/TFEB pathway.

## Abstract (web summary)

Congenital scoliosis is a complex genetic disorder characterized by vertebral malformation. Here, the authors demonstrate that loss of *dstyk* leads to scoliosis in zebrafish due to dysregulated biogenesis of notochord vacuoles and that DSTYK is required for lysosome biogenesis through mTORC1 regulation.

## Introduction

Congenital scoliosis (CS) is characterized by the lateral curvature of the spine with a Cobb angle >10 degrees and has an estimated prevalence approximately 1 in 1000 live births. It is mainly caused by defects in vertebral formation during embryogenesis^1,2^. The vertebral malformations in CS often consist of hemivertebrae, wedge-shaped vertebrae and vertebral fusions and they could occur throughout the spine and often impose severe spine deformity of the affected individuals^3^. CS may occur as an isolated case or as a part of a syndrome together with congenital defects in other organ systems^4,5^. Significant progress has been made in understanding the process of spine development and the main mechanisms of some CS^3,6,7^. Previous studies from animal models suggest that genetic factors are involved in the development of vertebral defects. To date, mutations disrupting multiple molecules and pathways such as Notch signaling pathway have been found to cause human CS^2,8–11^, however, the detailed genetic etiology and underlying mechanisms remain elusive.

Currently, patients with CS are important resources for the identification of causative genes for CS. However, owing to small pedigree size and locus heterogeneity, the genetic causes of most familial or sporadic cases of CS remain unknown. Developmental and genetic animal models are excellent tools for dissecting the biological origins and mechanism of scoliosis^12,13^. Teleosts have a natural susceptibility towards developing spinal deformity. Both genetic and environmental factors are associated with curvature in different fish species^14^. Teleost and human have similar biomechanical forces along the spine, which makes fish more susceptible to spinal curvatures^14–17^. Furthermore, spinal column morphology and structure of zebrafish are similar to those of humans. Several zebrafish mutants with a scoliosis-like phenotypes have recently been demonstrated to effectively mimic human CS and idiopathic scoliosis^15,18–22^. As the zebrafish has strong breeding ability, large-scale mutagenesis screening is an effective solution for finding mutations of genes causing CS in zebrafish. ENU is a highly potent mutagen which can induce point mutations. It is a commonly used for mutagenesis screening^23,24^.

In this study, we perform ENU mutagenesis screening and find a mutant zebrafish line with CS-like vertebral malformations. We confirm the responsible gene is *dstyk* using positional cloning and CRISPR/Cas9 approach. We find that *dstyk* mutant zebrafish have severe vertebral defects with fused and disorganized neural and haemal arches. The vertebral defects in *dstyk* mutants are caused by abnormal notochord development resulting from defects in biogenesis of notochord vacuoles. Further studies show that DSTYK is involved in the biogenesis of lysosome associated structures by promoting proper aggregation and fusion of late endosomal/lysosomal system in mammalian cells and zebrafish notochord cells. We reveal that *dstyk* mutation leads to defects in lysosome biogenesis and vacuole formation through repressing TFEB nuclear translocation via activating mTORC1. Inhibition of mTORC1 activity can partially rescue the defect in the biogenesis of notochord vacuole and scoliosis in *dstyk* mutants.

## Results

### *Smt* mutation leads to CS-like vertebral malformations

**Fig. 1:**
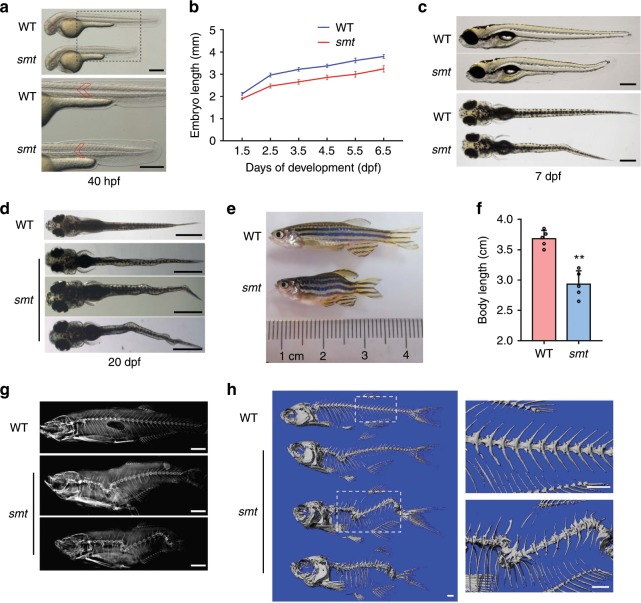
*Smt* mutation leads to CS-like vertebral malformations. **a** Bright-field images showing shortened somites and body length in *smt* mutant at 40 hpf. The bottom panel showed enlarged parts. Red line marks single somites. **b** Graph depicting the body length measurements of WT (blue line) and *smt* mutant (red line) from 1.5 dpf to 6.5 dpf, *n* = 20 independent embryos for WT and *smt* mutant. Data points represent average body length and error bars represent standard deviation. **c** Lateral (top) and dorsal views (bottom) showed slight wavy bending in *smt* mutant at 7 dpf. **d** At about 20 dpf, *smt* mutant showed different degrees of curve severity in dorsal view: mild (top), moderate (middle) and severe (bottom) curvature. **e** Whole mount image of 3-month-old *smt* mutant (bottom) and WT (top). *Smt* mutants had scoliosis and quantification of the body length were much shorter than WT (**f**), *n* = 5 independent zebrafishes for WT and *smt* mutant. ** *p* < 0.01. *p* values were determined by unpaired two-tailed Student’s *t*-test. X-rays (**g**) and three-dimensional micro-CT (**h**) revealed severe scoliosis and kyphosis in *smt* mutant (bottom). Boxed regions are magnified in the right panel in (**h**). Data are presented as mean ± SD. Scale bar represent 200 μm in (**a)**, 400 μm in (**c**), 1 mm in (**d** and **h**), 2 mm in (**d**). Source data are provided as a Source Data file.

To discover genes essential for spine development, we conducted an ENU-mediated genetic screen in zebrafish. We observed one mutant line, named *somite shorten* (*smt*^*cq75*^), whose somites were shortened but the average number had no significant difference compared with wild-type (WT) (Fig. 1a, Supplementary Fig. 1a, b). As early as 28 hours post fertilization (hpf), the body length was significantly decreased (Supplementary Fig. 1c, d). We measured the total lengths of *smt* mutants and WT siblings at different stages. The mutants showed a maximum 17.5% decrease in body length at 3 days post fertilization (dpf) (Fig. 1b). As early as 7 dpf, some mutants showed slight wavy bending compared to WT (Fig. 1c). At about 20 dpf, almost all the mutants showed dramatic lateral kinks at different degrees (Fig. 1d). As mutants grew to adult stage, from gross appearance and X-rays, they exhibited remarkable scoliosis and shorter than the WT siblings (Fig. 1e–g). Micro-CT examination further confirmed the severe scoliosis and kyphosis (Fig. 1h).

### *Smt* mutants encode alleles of *dstyk*

**Fig. 2:**
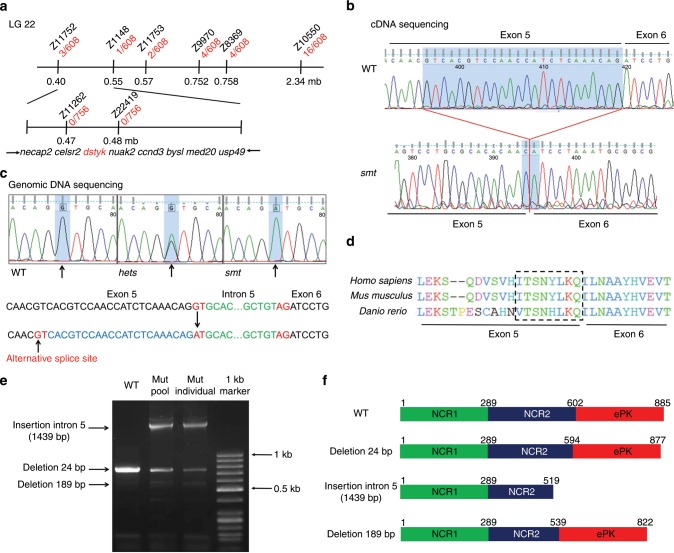
*Smt* mutants encode alleles of *dstyk*. **a** Genetic map of the candidate region on chromosome 22 (LG 22). Number of recombinants are depicted in red, and SSLP markers used for mapping are in black. **b** Sequencing of cDNA revealed that *smt* mutants had a 24 bp deletion at the end of exon 5 in *dstyk* coding sequence. **c** Genomic DNA sequencing revealed *smt* mutants had a *dstyk* G → A transversion in the first base of intron 5. Heterozygous *smt* is presented as hets. An alternative splice site is show in exon 5. **d** The mutation region of *dstyk* is evolutionally conserved across different species. Dotted box represents mutated amino acid. **e** PCR amplified the splicing mutation region (764 bp) of *dstyk* coding sequence showed more than three splicing types both in mutant pool and mutant individual. Arrows on the left indicate three different splicing types from agarose gel electrophoresis. **f** Diagramed DSTYK proteins structures of WT and three different predicted mutant proteins. WT DSTYK is comprised of two non-catalytic regions (NCR) and a eukaryotic protein kinase catalytic domain (ePK). Source data are provided as a Source Data file.

To identify the molecular lesion responsible for the phenotype, we used simple sequence length polymorphism (SSLP) to positionally clone the responsible gene^25^. The mutant allele *smt* was genetically mapped to chromosome 22 between the SSLP markers Z11752 and Z10550 (Fig. 2a). Through chromosomal walking and fine mapping, we further narrowed down the region between markers Z11752 and Z1148. This interval contains eight candidate genes, i.e., *necap2*, *celsr2*, *dstyk*, *nuak2*, *ccnd3*, *bysl*, *med20*, and *usp49* (Fig. 2a). We sequenced the entire coding sequence of these 8 genes of WT and *smt* mutants and identified that *dstyk* had a 24 bp deletion at the end of exon 5 (Fig. 2b). Further sequencing of the *dstyk* gene in mutants and sibling embryos using specific primers revealed a G → A transversion (IVS5 + 1G > A) in the first base of intron 5 (Fig. 2c). This splice-site mutation resulted in an in-frame deletion of eight amino acids in a domain that is highly conserved across different species (Fig. 2d). We amplified the coding region of mutant *dstyk* and cloned it into T vector. Sequencing revealed that there were more than three splicing types including insertion of intron 5 (1439 bp) and deletion of 24 bp or 189 bp, which leads to premature stop or amino acids deletion or insertion of Dstyk (Fig. 2e, f).

We performed rescue experiments using WT zebrafish *dstyk* mRNA and human *DSTYK* mRNA. Injection of 100 pg of WT mRNA at one-cell stage separately could significantly increase the body length of *smt* mutants at 4 dpf (Supplementary Fig. 2b, d). In addition, the defect vertebral formation and spinal curvature were alleviated after injection of either mRNA (Supplementary Fig. 2e). These data confirm that the *smt* mutant phenotype is caused by the loss-of-function mutation of *dstyk*.

**Fig. 3:**
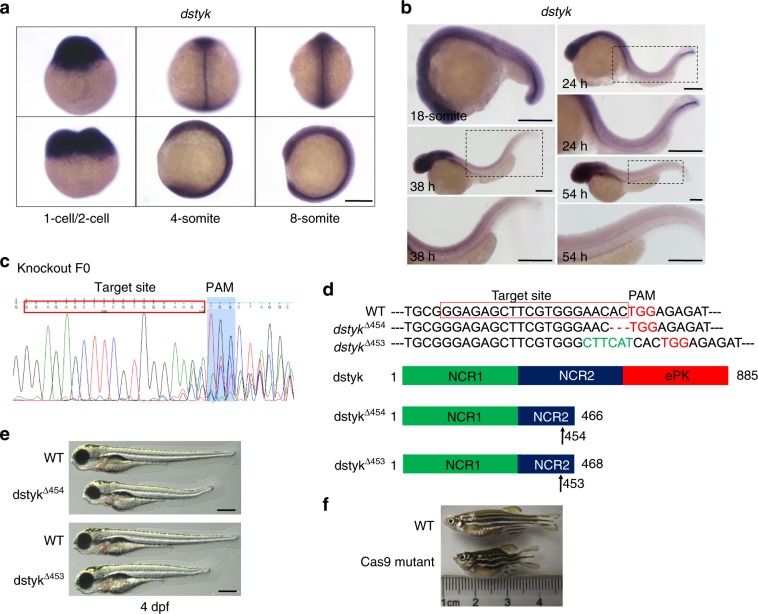
Expression pattern of *dstyk* and knock out *dstyk* by CRISPR/Cas9. **a** *Dstyk* ISH at 1-cell/2-cell, 4-somite and 8-somite stage. **b** From 18-somite, 24 hpf, 38 hpf to 54 hpf, notochordal expression of *dstyk* was dynamically changed as detected by ISH. **c** The Cas9 target site for knockout *dstyk* gene worked in knock out F0 generation. **d** Top panel showed the DNA sequences for WT and two Cas9 mutant lines. TGG (red) is the PAM sequence. Deletion is represented by a red dashed line and insertion is highlighted in green. Bottom panel diagramed DSTYK protein structures of WT and predicted mutants. Arrows indicate amino acid at the mutation site. **e** Bright-field images showing the WT and two Cas9 mutant lines at 4 dpf. **f** Cas9 mutant lines showed similar scoliosis phenotypes with *smt* mutant. Scale bar represent 200 μm in (**a** and **b**), 400 μm in (**e**).

We detected the expression pattern of *dstyk* by whole mount in situ hybridization (ISH), and found that *dstyk* was maternally and zygotically expressed (Fig. 3a). *Dstyk* transcripts were ubiquitously distributed at early embryonic stages but enriched in the notochord and head region at later stages (Fig. 3a, b). The notochordal expression of *dstyk* was dynamically changed, which was remarkable during somite stage, and then became stronger at the posterior notochord from 18-somite to 24 hpf. The expression of *dstyk* was weak but still observable at the notochord region from 38 hpf to 54 hpf, and finally was no longer detectable by 72 hpf (Fig. 3b). These observations imply that *dstyk* may play important role in the notochord development.

To further confirm if the defects in *dstyk* are responsible for the *smt* phenotype, we first used CRISPR/Cas9 system to knockdown *dstyk* (Fig. 3c). We identified two mutant lines with premature stop mutation caused by 2 bp frameshift deletion or 4 bp frameshift insertion (Fig. 3d). In F2 of these two mutant lines, approximately 25% of embryos had shortened somites and spinal deformity similar with that of *smt* mutants identified by ENU (Fig. 3e, f). Taken together, these data strongly support the *smt* mutant phenotype is caused by the loss-of-function mutation of *dstyk*.

### *Dstyk* mutation leads to abnormal notochord development

**Fig. 4:**
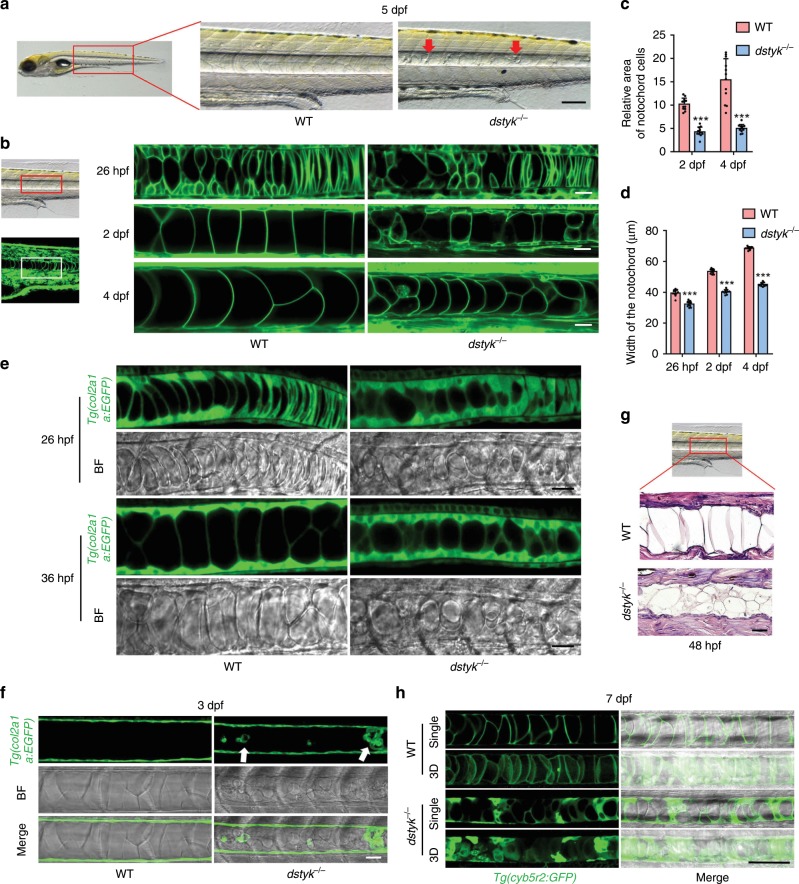
The phenotype of *dstyk* mutants in the notochord. **a** Bright-field images showing the WT and *dstyk* mutant at 5 dpf. Red arrow indicates the small granular structure accumulated in the notochord. **b** Live confocal images showing the notochord development of WT and *dstyk* mutant in *Tg(β-actin:ras-GFP)* transgenic background from 26 hpf to 4 dpf. All cell membranes were labeled by EGFP. **c** Quantification of relative area of single notochord cell for WT and *dstyk* mutant at 2 dpf and 4 dpf. *n* = 10 independent embryos for WT and *dstyk* mutant, 10 notochord cells were counted for each embryo. **d** Quantification of width of the notochord for WT and *dstyk* mutant from 26 hpf to 4 dpf. *n* = 12 independent embryos for WT and *dstyk* mutant. ***p < 0.001. *p* values were determined by unpaired two-tailed Student’s *t*-test. **e** Live confocal time-lapse images showing notochord development of WT and *dstyk* mutant in *Tg(col2a1a:EGFP)* transgenic background (left) and bright-field (right) at 26 hpf and 36 hpf. **f** Live confocal images of WT and *dstyk* mutant in *Tg(col2a1a:EGFP)* transgenic background at 3 dpf. White arrow indicates ectopic Col2a1a positive cells in the notochord in the mutants. **g** Hematoxylin and eosin staining of longitudinal sections of the notochord in WT and *dstyk* mutant at 48 hpf. **h** Confocal images showing the notochord of WT and *dstyk* mutants in *Tg(cyb5r2:GFP)* transgenic background at 7 dpf. All notochord cells were labeled by EGFP. Data are presented as mean ± SD. Scale bar represent 20 μm in (**b**, **e**, **f** and **g**), 100 μm in (**a** and **h**). Source data are provided as a Source Data file.

In consideration of the characteristic expression of *dstyk* in notochord and the involvement of notochord in the spine development^6,26^, we hypothesize that the scoliosis phenotype of *dstyk* mutants is due to the notochord maldevelopment. Notochord in WT was a rod-like structure like a stack of pennies^26^, but in the mutants, there were many small granular structures accumulated within the notochord at 5 dpf (Fig. 4a). To observe the dynamic changes in notochord defects, we generated *dstyk* mutants in the background of *Tg(β-actin:ras-GFP)* transgene^27^, in which all cell membranes were labeled by EGFP. Confocal microscopy revealed that the stacked notochord cells in WT were gradually inflated along the anterior-posterior axis at 26 hpf^26^, and finally became well arranged inflated cells with large vacuoles in 2–4 dpf (Fig. 4b). However, in the mutants, the notochord cells were unequally inflated with irregular arrangement along the anterior-posterior axis. In particularly, most notochord vacuoles in mutants cannot be fully expanded to their maximum volume, leading to collapse of the EGFP labeled notochord cell membranes (Fig. 4b). Compared to WT, the sizes of the notochord cells were significantly decreased in mutants in 2 and 4 dpf (Fig. 4c), resulting in significantly decreased the width and length of the notochord (Fig. 4d).

Furthermore, we generated *dstyk* mutants in the background of *Tg(col2a1a:EGFP)* transgene^28^, which can label both notochord cells and notochord sheath cells at early embryonic stage, and after 2 dpf only label notochord sheath cells. At about 24–48 hpf in the WT, some stacked flat cells developed into vacuolated notochord cells, while other flat cells differentiated into notochord sheath cells surrounding the notochord. However, time series of live cell imaging revealed that the mutant notochord cells cannot be fully inflated (Fig. 4e). Furthermore, in 3 dpf, Col2a1a expression was only present in the WT notochord sheath cells, but there were some ectopic Col2a1a expression within the mutant notochord (Fig. 4f). Hematoxylin-eosin staining also revealed that, at 48 hpf the mutant notochord was composed of smaller vacuolated cells with relatively more cytoplasm and many smaller vesicle-like structures that were not fully developed into mature vacuole in contrast to the WT (Fig. 4g). We also found various sized and disorderly arranged notochord cells in mutants at 7 dpf by using a transgenic line *Tg(cyb5r2:GFP)* that labels notochord cells^29^ (Fig. 4h).

We further found increased proliferation of notochord cells in the mutants using Edu labeling (Supplementary Fig. 3a, c). Consistently, there were more DAPI labeled nucleus in the mutant notochord (Supplementary Fig. 3d). TUNEL assay revealed that there was no significant difference in the apoptosis between the notochord of WT and mutants (Supplementary Fig. 3b). These data revealed that there was increased proliferation in the notochord of *dstyk* mutants, leading to more cells in the notochord than that of the WT, although the sizes of mutant cells were smaller. These results suggest that loss-of-function of Dstyk leads to dysregulated notochord development mainly due to the incomplete vacuole inflation of notochord cells.

### *Dstyk* mutation inhibits notochord vacuoles biogenesis

**Fig. 5:**
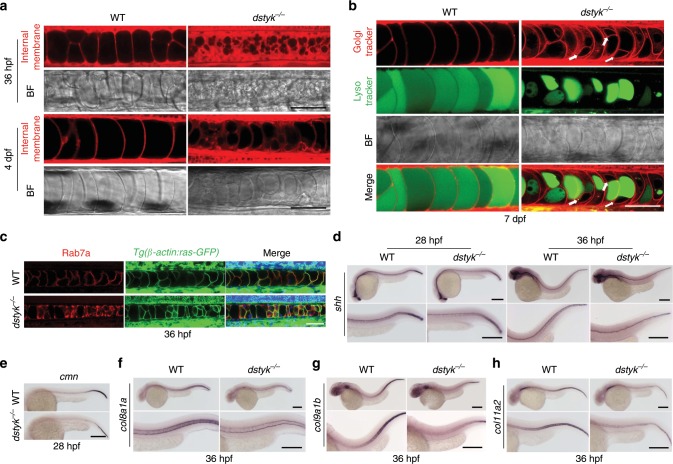
*Dstyk* gene mutation leads to abnormal vesicle trafficking and dysregulated notochord vacuoles biogenesis. **a** Live confocal images of notochord for WT and *dstyk* mutant in bright-field and staining with MED at 36 hpf (top) and 4 dpf (bottom). **b** Live confocal images of notochord for WT and *dstyk* mutant dyed with Golgi tracker BODIPY TR Ceramide and LysoTracker Green at 7 dpf. Note that every notochord cell in the mutant was composed of one LysoTracker Green labeled vacuole (green) and at least one round cystic structure (white arrows) that can be labeled by Golgi tracker. **c** Confocal images of immunofluorescent staining of WT and *dstyk* mutants for Rab7a at 36 hpf in the background of *Tg(β-actin:ras-GFP)*. **d** ISH of *shh* at 28 hpf (left) and 36 hpf (right) of WT and *dstyk* mutants. Magnified images in the bottom column. **e** ISH of *cmn* at 28 hpf of WT and *dstyk* mutants. **f**–**h** ISH of *col8a1a* (**f**), *col9a1b* (**g**) and *col11a2* (**h**) at 36 hpf of WT and *dstyk* mutants. Magnified images in the bottom column. Scale bar represent 50 μm in (**a**–**c**), 200 μm in (**d**–**h**).

Zebrafish notochord vacuoles are identified as specialized lysosome-related organelles (LROs) that have important function in body axis elongation and spine morphogenesis^26^. We used multiple vital dye trackers to determine whether the deformity of mutant notochord is related to the abnormal vacuole formation. Firstly, we used BODIPY TR methyl ester dye (MED) to label internal membranes^30^. Confocal imaging showed that the vacuoles of *dstyk* mutants were fragmented and generally smaller than that of WT at 2 dpf (Fig. 5a). Live time-lapse imaging of vacuole expansion from 20 to 30 hpf revealed that notochord vacuoles were well inflated in WT, during which the MED labeled internal membranes gradually fused (Supplementary Movie 1). In contrast, in mutants, majority of small vesicle structures did not fuse together to form large notochord vacuoles (Supplementary Movie 2). There were more cytoplasm and subcellular structures labeled by MED in the mutant notochord cells, indicating that vacuole formation was partially disrupted at the vesicle trafficking stage in the mutants. In addition, we found that the defects of notochord vacuole biogenesis of *smt* mutants were alleviated after zebrafish *dstyk* mRNA or human *DSTYK* mRNA injection (Supplementary Fig. 2a, c).

Notochord vacuoles inflation could receive contributions from biosynthetic trafficking through the ER to Golgi complex transport^26^. The formation and maintenance of vacuole requires post-Golgi late endosomal trafficking regulated by the vacuole-specific Rab32a and H^+^-ATPase–dependent acidification^26^. To determine which phase was blocked during the vesicle trafficking in *dstyk* mutants, we used Golgi tracker BODIPY TR Ceramide, and LysoTracker Green to label acidic notochord vacuoles (Fig. 5b). The confocal images revealed that large vacuoles labeled with green LysoTracker occupied almost the whole notochord cells, which arranged into a single row along anterior-posterior axis of notochord in WT at 7 dpf. However, the mutant vacuoles were smaller with multiple shapes, and only partially occupied the cytoplasm. The other regions of notochord cells were mainly labeled by Golgi tracker that largely labels Golgi apparatus. Interestingly, most mutant notochord cells were composed of various sized vacuole and at least one round cystic structure that can be labeled by Golgi tracker (Fig. 5b). LysoTracker Green and BODIPY TR Ceramide staining of *dstyk* mutants in the background of *Tg(β-actin:ras-GFP)* showed similar results (Supplementary Fig. 4a).

It has been reported that notochord vacuoles are specialized post-Golgi structures whose biogenesis and maintenance require late endosomal trafficking^26^. We hypothesized that the round cysts in *dstyk* mutants are the sorting endosome or late endosome, which are normally derived from Golgi complex to form vacuoles. To test this hypothesis, we detected the expression of late endosome marker Rab7a at 36 hpf in the background of *Tg(β-actin:ras-GFP)* and revealed that Rab7a had weak expression in the WT notochord, but were strongly expressed in the mutant notochord (Fig. 5c). Double immunofluorescent staining of Rab7a and Col2 of mutants showed similar results (Supplementary Fig. 4b). These data indicate that late endosomal trafficking to form vacuoles was partially blocked in the *dstyk* mutant.

The notochord is an important source of signals for patterning of adjacent tissues during development^31^. Abnormalities in the vesicle trafficking may lead to dysregulated secretion of signaling factors^32^. We found that the expression of a major secreted signaling factor, *sonic hedgehog* (*shh*) was decreased in the mutant notochord at 28 and 36 hpf (Fig. 5d). In addition, we found that *calymmin* (*cmn*), a notochord sheath-associated extracellular matrix (ECM) protein^33^, was decreased in the mutant notochord at 28 hpf (Fig. 5e). Furthermore, we found that the RNA levels of several collagens, the most abundant ECM proteins, including *col8a1a*, *col9a1b*, *col11a2* were also decreased in the mutant notochord at 36 hpf (Fig. 5f–h). Together, these data suggest that mutation of *dstyk* leads to notochord maldevelopment resulting from abnormal vesicle trafficking and blocked vacuole formation, and dysregulated notochord function.

### Ultrastructure of notochord and sheath in *dstyk* mutants

**Fig. 6:**
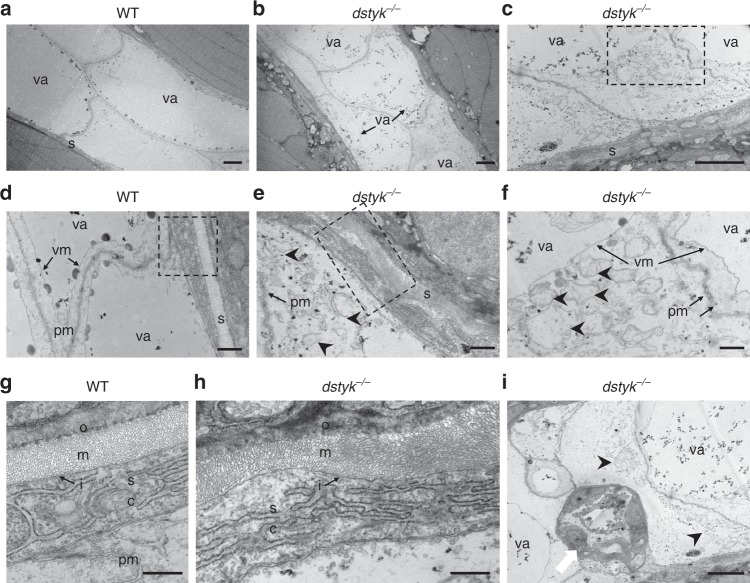
Ultrastructure of the notochord and notochord sheath in WT and *dstyk* mutant. **a**–**f** The transmission electron micrographs of the notochord in WT (**a**, **d**) and *dstyk* mutant (**b**, **c**, **e** and **f**) at 48 hpf. Note that the vacuoles were smaller in the *dstyk* mutant, and there were many scatteredly distributed small vesicles that were wrapped by single layer membrane in the cytoplasm of *dstyk* mutant. **g**, **h** The ultrastructure of the notochord sheath in the WT (**g**) and *dstyk* mutant (**h**). Note the straight, well-organized sheath layer in the WT and wavy, disordered organization of the medial layer in the *dstyk* mutant. **i** Ultrastructure of the notochord showed not well vacuolated epithelia-like cells (white arrow) aggregated in *dstyk* mutant. **f**, **g** and **h** were magnified image of the dotted boxes in **c**, **d** and **e**, respectively. Black arrowheads indicate many small vesicles in the mutant cytoplasm. All images are shown longitudinal sections. The scale bars represent (**a**–**c**, and **i**) 5 µm, (**d**–**f**) 1 µm and (**g**, **h**) 500 nm. va, vacuole; s, notochord sheath; sc, sheath cell; pm, plasma membrane; vm, vacuole membrane; i, inner laminin-rich layer, m, medial layer, and o, outer collagen-rich layers of the notochord sheath.

To further investigate the detailed defects in ultrastructure of the notochord, transmission electron microscopy (TEM) analysis was performed. TEM revealed that the WT notochord at 48 hpf was consisted of two rows of large vacuolated notochord cells and their cytoplasm was squeezed by the expanded vacuole to a very small area close to the cell membrane^26^ (Fig. 6a–d). In contrast, mutant notochord cells were much smaller with an irregular arrangement. The vacuoles in mutant cells just occupied a small area of the cytoplasm, while unoccupied area contained many scatteredly distributed small vesicles wrapped by the single layer of membrane (Fig. 6b–f). The small vesicle structure in mutant cells may be the post-Golgi structures or late endosome^26^, as observed many Rab7a positive structures in these regions.

Notochord sheath is a tri-laminar extracellular sheet composed of an inner laminin-rich basement membrane, collagen-rich medial layer and an outer layer composed of collagen and elastin fibers^34^. Since the formation of notochord sheath is closely linked to the differentiation of notochord cells^29,35^, we used TEM to analyze the ultrastructure of the mutant notochord sheath. At 48 hpf, WT sheath is a straight and well-organized three layers structure with (Fig. 6d–g). However, the mutant sheath exhibited a wavy structure with disordered organization of sheath layers. Some regions of mutant sheath became thinned, while others had much thicker sheath layers especially the middle layer (Fig. 6e–h). Taken together, these results strongly suggest that *dstyk* mutation blocked notochord vacuole biogenesis leading to malformed notochord sheath.

### *Dstyk* regulates axial skeleton segmentation and formation

**Fig. 7:**
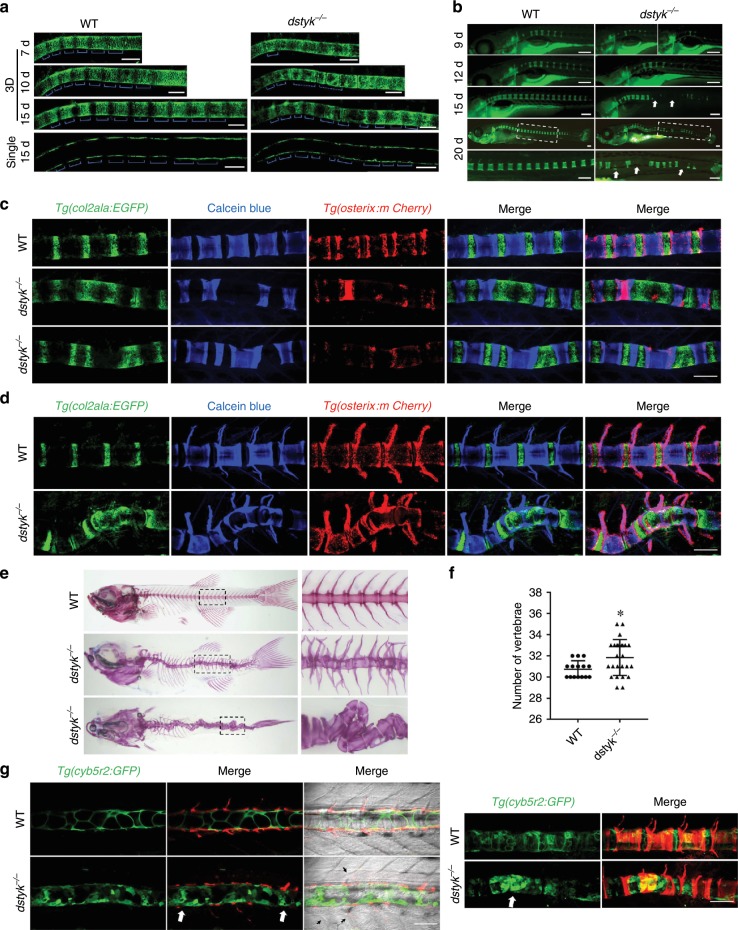
*Dstyk* regulates axial skeleton segmentation and spine formation. **a** Confocal images showing the notochord sheath cells of WT (left) and *dstyk* mutants (right) in *Tg(col2a1a:EGFP)* transgenic background from 7 to 15 dpf. Top three panel show the 3D view and bottom panel shows the single layer. The blue brackets indicate *col2a1a* positive domains. **b** Live images of Calcein staining for WT (left) and *dstyk* mutants (right) from 9 to 20 dpf. Boxed regions at about 20 dpf are magnified in the bottom panel. Note that wedge-shaped and mineralization defect vertebrae (white arrows) were shown in *dstyk* mutants. **c**, **d** Confocal images showing Calcein blue staining of *Tg(col2a1a:EGFP)*;*Tg(osterix:mCherry)* double transgenic fish of WT and *dstyk* mutants at 20–25 dpf (**c**) and at 30–40 dpf (**d**). Note the disordered notochord sheath segmentation and curvature of the spine. **e** Lateral (middle) and dorsal view (bottom) of vertebral structure stained with Alizarin red for *dstyk* mutant zebrafish at about 1 month of age. WT (top) is shown the lateral view. Boxed regions are magnified in the right panel. **f** Graph depicting the counted number of vertebrae of WT (*n* = 15 independent embryos) and *dstyk* mutants (*n* = 25 independent embryos), *p < 0.05. *p* values were determined by unpaired two-tailed Student’s *t*-test. **g** Confocal images show the notochord cell of WT and *dstyk* mutants in *Tg(cyb5r2:GFP)* transgenic background and in vivo skeletal staining of centra with Alizarin red at 20–25 dpf. Left panel shows the single layer and right panel shows the 3D view. White arrows show the severe vacuole biogenesis defect. Black arrowheads show somite boundaries in mutants were not as sharp and straight as those in WT. Data are presented as mean ± SD. Scale bar represent 100 μm in (**a**, **c**, **d** and **g**), 200 μm in (**b**). Source data are provided as a Source Data file.

To explore how the *dstyk* mutants develop scoliosis at adult stages, we analyzed the process of axial skeleton segmentation, vertebral mineralization and spine formation. At early stages, *Tg(col2a1a:EGFP)* uniformly labeled all notochord sheath cells (Fig. 4f). But from about 7 to 15 dpf, we found that *Tg(col2a1a:EGFP)* generated an alternating pattern of *col2a1a* positive and negative domains in an anteroposterior direction in WT sheath cells. But in *dstyk* mutant, the segmented pattern was disrupted with irregularly distributed *col2a1a* positive domains (Fig. 7a). Using vital fluorescent Calcein green staining to detect vertebral mineralization, we found that the mineralization of segmented chordacentra occurred sequentially in an anteroposterior direction in WT from 9 to 20 dpf, but the formation of segmented chordacentra was disordered and delayed in *dstyk* mutants (Fig. 7b). As segmentation of the zebrafish axial skeleton relies on notochord sheath cells^36,37^, we generated *dstyk* mutants in the *Tg(col2a1a:EGFP)*;*Tg(osterix:mCherry)* double transgenic background that report notochord sheath cells and osteoblasts respectively to observe the mineralization of segmented chordacentra and the formation of vertebral body. Calcein blue staining revealed apparently disordered chordacentra formation and wedged, incompletely or irregularly sized chordacentra with curved notochord sheath in *dstyk* mutants at 20–25 dpf (Fig. 7c). With the mineralization of notochord sheath, osteoblasts are subsequently recruited to the chordacentra to form mature vertebral bodies. We found that the curvature of spine was gradually aggravated with abnormal vertebrae formation in mutants at 30–40 dpf (Fig. 7d). Alizarin red staining further showed that the body axis was distorted with deformed vertebral body and highly disorganized neural and haemal arches (Fig. 7e). As alteration of notochord sheath segmentation may alter the vertebrae number^36,37^, we found that in contrast to number ranged from 30 to 32 in WT, the vertebrae number in mutants ranged from 29 to 35 leading to increased average number (Fig. 7f). In addition, we generated *dstyk* mutants in the *Tg(cyb5r2:GFP)* transgenic background to label notochord cells. Interestingly, we found that the location of chordacentra mineralization defects and bending region in the spine mostly was the sites with severe vacuole biogenesis defect (Fig. 7g), indicating that abnormal notochord vacuole development is involved in the chordacentra mineralization and curvature of spine. We also found that some somite boundaries in mutants were not as sharp and straight as those in WT (Fig. 7g). Together, these data suggest that *dstyk* mutation leads to abnormal axial skeleton segmentation and defect spine formation, eventually leading to scoliosis.

### DSTYK is involved in lysosome biogenesis

**Fig. 8:**
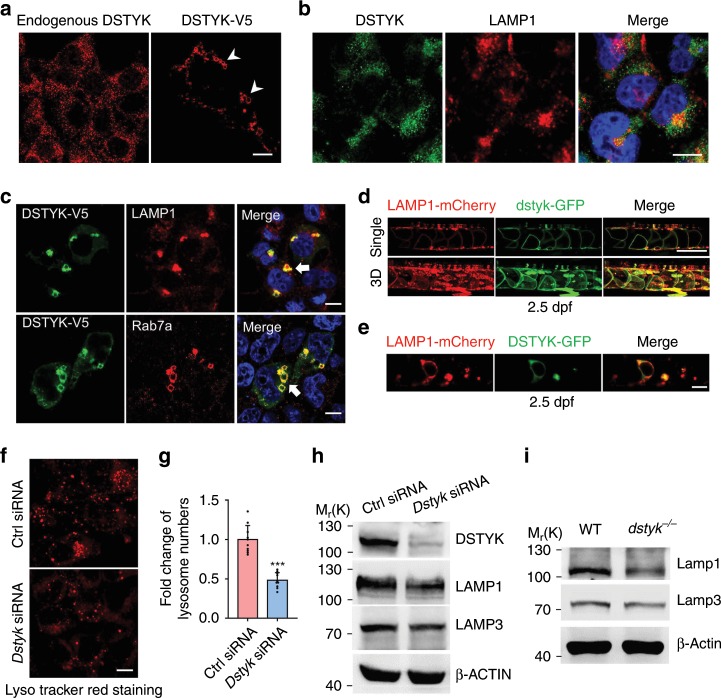
DSTYK is involved in lysosome biogenesis. **a** Representative images of immunofluorescent staining for endogenous DSTYK (left) and transfected with exogenous DSTYK-V5 (right) in COS-7 cells. White arrowheads indicate large ring-like DSTYK-positive structures. **b** Double immunofluorescent staining of endogenous DSTYK with LAMP1 in COS-7 cells. **c** COS-7 cells transfected with DSTYK-V5 and double immunofluorescent staining of V5 with LAMP1 (top) and Rab7a (bottom). White arrows indicate large ring-like structures co-localization of DSTYK-V5 with LAMP1 and Rab7a. **d** Confocal images show the 2.5 dpf mosaic transgenic fishes expressing dstyk-GFP and LAMP1-mCherry *(Tg(hsp70l:dstyk-GFP)*;*Tg(hsp70l:LAMP1-mCherry))*. Top panel show the single layer and bottom panel showed the 3D view. **e** Confocal images show the single layer of 2.5 dpf mosaic transgenic fishes expressing DSTYK-GFP and LAMP1-mCherry *(Tg(hsp70l:DSTYK-GFP)*;*Tg(hsp70l:LAMP1-mCherry))*. Heat shocking at 72 hpf and imaging at about 78 hpf. **f** Representative images of LysoTracker Red staining of lysosome after transfected with control siRNA (top) and *Dstyk* siRNA (bottom) in COS-7 cells. **g** Quantifications of lysosome numbers after transfected with control siRNA or *Dstyk* siRNA. *n* = 10 independent views from three independent experiments, five cells were counted for each view. ***p < 0.001. *p* values were determined by unpaired two-tailed Student’s *t*-test. **h** Immunoblotting of LAMP1 and LAMP3 in COS-7 cells transfected with control siRNA and *Dstyk* siRNA. **i** Immunoblotting of Lamp1 and Lamp3 in WT and *dstyk* mutant zebrafish. Data are presented as mean ± SD. Scale bar represent 10 μm in (**a**–**c**), and (**f**), 100 μm in (**d**, **e**). Source data are provided as a Source Data file.

Zebrafish notochord vacuole is a type of specialized LROs and shares many similar features with lysosomes including similar signaling pathways regulating their formation^38^. Therefore, DSTYK may also participate in the formation of lysosomes in mammals through similar biogenesis mechanism. DSTYK has been found distributed in cytoplasm^39^ or cell membranes^40^. We found that endogenous DSTYK was mainly present in tiny puncta throughout the cytoplasm in COS-7 cells (Fig. 8a). Immunofluorescence revealed some overlapping between endogenous DSTYK and the late endosomal/lysosomal marker LAMP1 (Fig. 8b). When DSTYK-V5 fusion protein is overexpressed in COS-7 cells, most of the DSTYK-V5 protein was fused, forming vesicle structures with variable sizes (Fig. 8a). In addition, LAMP1 labeled lysosomal compartments, which are normally distributed as dot like structure in perinuclear region, were fused and aggregated to form large vesicles after DSTYK overexpression (Fig. 8c). The LAMP1 signals were almost completely co-localized with DSTYK. Rab7a is normally present in the late endosome and throughout the cytoplasm as dot like structure. We demonstrated that Rab7a labeled structure was aggregated and fused to become large ring-shaped vesicles in the perinuclear region in response to DSTYK overexpression (Fig. 8c). The sizes of some ring-shaped vesicles were up to 3–5 μm. Meanwhile, the Rab7a was also co-localized with DSTYK like LAMP1. These data indicate that mammalian DSTYK is mainly localized to late endosomal/lysosomal compartments and DSTYK overexpression promotes the fusion of late endosomal/lysosomal system, inducing late endosomes/lysosomes enlargement.

To examine the intracellular localization of *dstyk* in notochord cells, we generated two mosaic transgenic fishes expressing zebrafish Dstyk tagged by GFP and human LAMP1 tagged by mCherry under the control of a heat shock protein promoter. As Ellis et al. have reported that Lamp1-RFP labels vacuole membrane as well as lysosomes^26^, we found that Dstyk-GFP had remarkable colocalization with LAMP1-mCherry on the vacuole membrane and lysosomes in notochord cells (Fig. 8d). In addition, we generated mosaic transgenic fish expressing human DSTYK tagged with GFP under the control of a heat shock protein promoter. Live confocal imaging revealed a similar colocalization of DSTYK-GFP with LAMP1-mCherry on the vacuole membrane and lysosomes (Fig. 8e). These in vivo studies indicate that Dstyk has consistent localization as those obtained in cultured human cells.

**Fig. 9:**
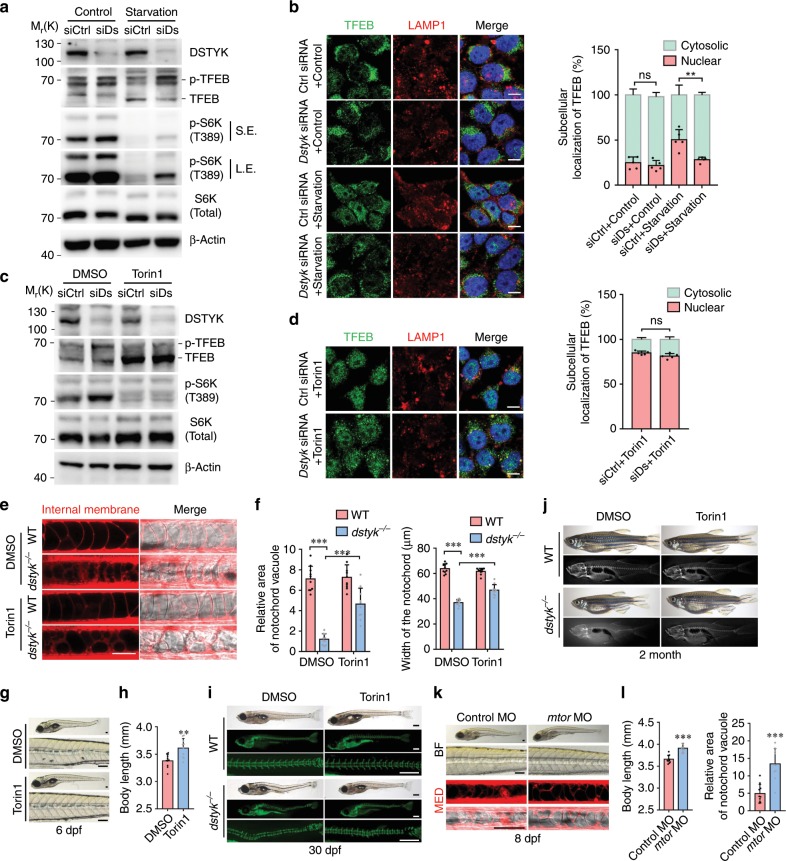
*Dstyk* regulates lysosome biogenesis through mTORC1/TFEB pathway. **a**, **c** Immunoblotting of endogenous TFEB, p-TFEB (phosphorylated TFEB) and p-S6K(T389) in COS-7 cells transfected with control siRNA (siCtrl) or *Dstyk* siRNA (siDs), **a** cells without starvation (Control) or starved for 2 h. S.E./L.E., Shorter exposure/longer exposure. **c** cells treated with DMSO or Torin1 (1 µM) for 3 h. **b**, **d** Immunofluorescence for TFEB and LAMP1 in COS-7 cells transfected with control siRNA or *Dstyk* siRNA, **b** cells without starvation (Control) or starved for 2 h, **d** cells treated with Torin1 (1 µM) for 3 h. Quantifications of subcellular localization of endogenous TFEB (a percentage of total immunostaining colocalized with nuclear or cytosolic staining) was shown in right panel. *n* = 5 independent experiments, each experiment counted 10 cells. **p < 0.01. **e**–**j** WT and mutant were treated with DMSO or 500 nM Torin1 from 20 to 30 hpf. Vacuole revealed by MED staining (left) at 48 hpf in (**e**). Quantification of relative area of notochord vacuole (left) (*n* = 10 indepe*n*dent embryos, each embryo counted 10 vacuoles) and notochord width (right) (*n* = 10 indepe*n*dent embryos) at 48 hpf in (**f**). ***p < 0.001. Bright-field images showing the 6 dpf mutants treated with DMSO (top) or Torin1 (bottom) in (**g**). Quantification of the body length of mutants in (**h**). *n* = 10 independent embryos. **p < 0.01. Calcein staining and bright-field images of about 30 dpf WT (top) and *dstyk* mutants (bottom) in (**i**). Whole mount and X-rays images of 2-month-old WT (top) and *dstyk* mutants (bottom) in (**j**). **k** Bright-field (top) and MED staining (bottom) images showing the 8 dpf *dstyk* mutants after injected with control (left) and *mtor* MO (right). **l** Quantification of the body length (left) (*n* = 10 independent embryos) and relative area of notochord vacuole (right) (*n* = 10 independent embryos, each embryo counted 10 vacuoles) after control and *mtor* MO injection. ***p < 0.001. *p* values in (**b**, **d** and **f**) determined by two-way ANOVA test, *p* values in (**h** and **l**) determined by unpaired two-tailed Student’s *t*-test. Data are presented as mean ± SD. Scale bar represent 10 μm in (**b** and **d**), and 50 μm in (**e**), 100 μm in (**g** and **k**), 500 μm in (**i**). Source data are provided as a Source Data file.

To further assess the effects of DSTYK deficiency on lysosome biogenesis, we evaluated the lysosome numbers in COS-7 cells with *Dstyk* siRNA knockdown using lysosome-specific dye LysoTracker Red. We found that lysosome numbers and fluorescence intensity were reduced after *Dstyk* knockdown (Fig. 8f, g), and decreased levels of LAMP1 and LAMP3 (Fig. 8h, Fig. 9b). Similar results were also observed in HeLa cells (Supplementary Fig. 5a). Furthermore, the protein levels of Lamp1 and Lamp3 were decreased in *dstyk* mutants as well (Fig. 8i). These data indicate that DSTYK is essential for the normal biogenesis and maintenance of the lysosomal compartment in mammalian cells.

### *Dstyk* regulates lysosome biogenesis via mTORC1/TFEB pathway

The biogenesis of lysosome and LROs can be triggered by the transcription factor TFEB^41,42^. Under starvation or lysosomal stress conditions, TFEB is dephosphorylated and subsequently translocated to the nucleus to activate its target genes^43–45^. We investigated whether DSTYK acts through TFEB to modulate lysosome and vacuole biogenesis. To assess TFEB phosphorylation, we compared the electrophoretic mobility of endogenous TFEB in the cells transfected with control and *Dstyk* siRNA with and without starvation. We found that most TFEB were in phosphorylation state in non-starved cells transfected with control siRNA or *Dstyk* siRNA, although the electrophoretic mobility shifts were a little faster in control siRNA cells (Fig. 9a). Upon starvation, most TFEB was dephosphorylated in control siRNA cells, while in those *Dstyk* siRNA cells most TFEB were still in phosphorylation state (Fig. 9a). In addition, TFEB nuclear translocation in response to starvation was markedly reduced in *Dstyk*-siRNA cells compared to control cells, without starvation the TFEB nuclear translocation had no obvious change between control and *Dstyk*-deficient COS-7 cells (Fig. 9b). Similar results were also observed in HeLa cells by immunostaining (Supplementary Fig. 5a). These data indicate that DSTYK inhibition may promote the phosphorylation of TFEB and subsequently repress its nuclear translocation, inhibiting the biogenesis of lysosome and LROs^45^.

mTORC1 has been found to phosphorylate TFEB at its Ser142 and Ser211 leading to inhibited TFEB nuclear translocation^43,44^. We therefore examined whether DSTYK regulate of TFEB phosphorylation through mTORC1. We found that in the presence of sufficient nutrients, mTOR had remarkable co-localization with DSTYK-V5 in COS-7 cells (Supplementary Fig. 6a). Western blot analysis revealed that the phosphorylation of p70 ribosomal S6 kinase (S6K), a mTORC1 downstream substrate, was elevated in *Dstyk*-deficient COS-7 cells both in starved and non-starved status in contrast to the control cells (Fig. 9a–c). In addition, during starvation, most mTOR was located in tiny puncta throughout the cytoplasm in control siRNA transfected cells, whereas in *Dstyk* siRNA cells, mTOR was moved to small vesicular structures and co-localized with LAMP1 (Supplementary Fig. 6b), similar to those observed in cells with amino acid-induced activation of mTORC1^46^. These data suggest that knockdown of *Dstyk* activates mTORC1 and thus repress TFEB nuclear translocation. Next, we sought to determine whether mTOR1 inhibitor Torin1 affects the phosphorylation and nuclear translocation of TFEB and lysosome biogenesis in *Dstyk* knockdown cells. We found that in *Dstyk*-deficient COS-7 cells, treatment with Torin1 caused a molecular weight shift of TFEB similar to that exhibited in control cells (Fig. 9c). Immunofluorescence results revealed that majority of TFEB was translocated to the nucleus following Torin1 treatment (Fig. 9d). The protein levels of LAMP1 were increased both in *Dstyk*-deficient cells and control cells after Torin1 treatment (Fig. 9d). Similar results were also observed in HeLa cells (Supplementary Fig. 5a, b).

Next, we sought to determine whether Dstyk regulate Tfeb and mTOR in zebrafish. We found that the levels of *tfeb* and several *tfeb* target genes involved in the lysosomal pathway were significantly downregulated in *dstyk* mutants (Supplementary Fig. 7a). ISH revealed that the expression of *lamp1b*, a *tfeb* target gene, was significantly decreased in the notochord of *dstyk* mutants (Supplementary Fig. 7b). Lamp1 is a glycoprotein responsible in part for maintaining lysosomal integrity, pH and catabolism, which is also involved in the lysosome biogenesis^47^. We found that the levels of mTORC1 downstream substrate phosphorylated S6 ribosomal protein (pS6) were significantly increased in the notochord of *dstyk* mutants in *Tg(β-actin:ras-GFP)* background (Supplementary Fig. 7c, e), while the level of S6 ribosomal protein (S6) had no significant change (Supplementary Fig. 7d, f). Then, we asked whether mTORC1 inhibition could rescue the phenotypes of *dstyk* mutants. Torin1 treatment during notochord cell vacuole formation from 20 to 30 hpf could partially rescue the defects in notochord vacuole biogenesis, the decreased width of notochord and the decreased body length (Fig. 9e–h). Calcein skeletal staining revealed that vertebral mineralization and spinal curvature were partially rescued after Torin1 treatment at about 30 dpf (Fig. 9i). In addition, the degree of scoliosis in Torin1 treated mutants was partially alleviated in contrast to DMSO-treated mutants in adulthood (Fig. 9j). We also found that injection of a splicing block morpholino (MO) targeting the kinase domain of mtor can partially rescue the defect of notochord vacuole biogenesis and the decreased body length in *dstyk* mutants. In addition, the body curvature was also alleviated after *mtor* knockdown at 8 dpf. (Fig. 9k, l). Taken together, our in vivo and in vitro data indicate that *Dstyk* mutation could lead to defects in vacuole formation and lysosome biogenesis, partially through the resultant mTORC1 activation-mediated repression of TFEB nuclear translocation.

## Discussion

**Fig. 10:**
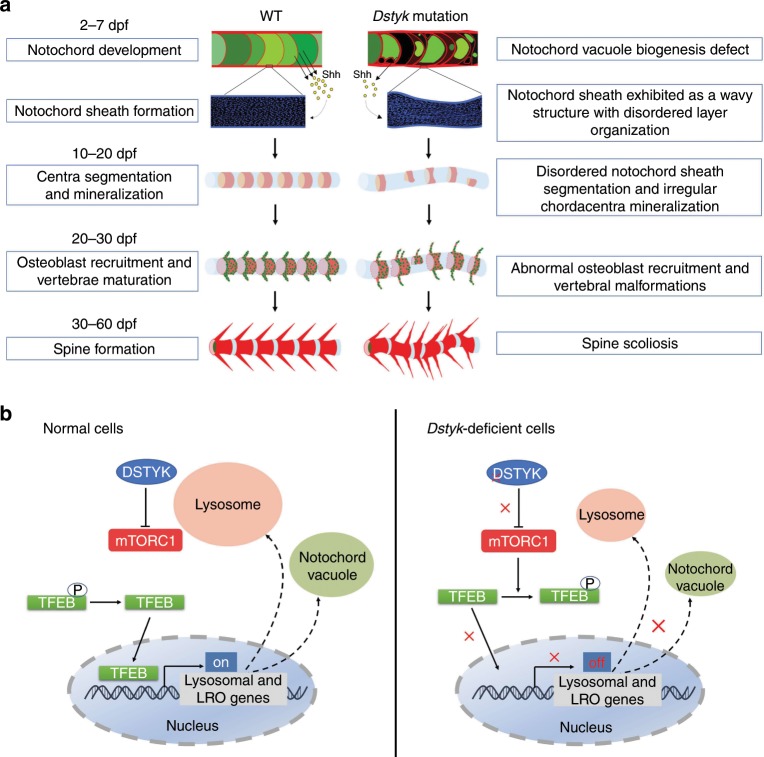
Schematic diagram for vertebral column development and proposed model for the regulation of mTORC1/TFEB pathway by DSTYK. **a** The development process of notochord and vertebral column in WT and *dstyk* mutant (See also in “Discussion”). **b** Model depicting the proposed mechanistic regulation of TFEB by DSTYK. In normal cells, DSTYK could inhibit the activity of mTORC1, resulting in nuclear translocation of non-phosphorylated TFEB and promoting lysosomal and LRO genes transcription. In *Dstyk*-deficient cells, mTORC1 is activated and phosphorylates TFEB, resulting in reduced TFEB nuclear translocation and inhibition of lysosomal and LRO genes transcription.

In this study, using ENU mutagenesis screening, we found a mutation in *dstyk* can lead to CS-like vertebral malformations in zebrafish. *Dstyk* mutants have severe vertebral defects with fused and disorganized neural and haemal arches. We provide further evidence suggesting that the vertebral defects in *dstyk* mutants are related to the wavy and malformed notochord sheath and abnormal axial skeleton segmentation resulting from defect biogenesis of notochord vacuole (Fig. 10a). We found that DSTYK regulates lysosome biogenesis by promoting proper aggregation and fusion of late endosomal/lysosomal system in mammalian cells. Defect vacuole formation and lysosome biogenesis observed in *dstyk* mutants may be related to the repression of TFEB nuclear translocation resulting from activation of mTORC1 (Fig. 10b).

DSTYK is a widely expressed in vertebrates^39,48^. We found that *dstyk* had relatively strong expression in zebrafish notochord during its formation and development. Consistently, DSTYK also showed relatively specific expression in the mouse notochord (Supplementary Fig. 8). These data indicate that the expression of DSTYK in notochord is conserved between zebrafish and mammals. Considering the essential role of notochord in spine development^26,49^, we speculate that the scoliosis in *dstyk* mutants may be due to defects in notochord development. Notochord vacuoles are required for the body axis elongation and therefore the proper spine morphogenesis^26^. The notochord vacuoles are identified as specialized LROs^26^, which was confirmed by the in vivo labeling of the vacuoles with vital dye LysoTracker (Fig. 6b). Using live cell imaging and TEM we found that the biogenesis of notochord vacuole was disrupted in *dstyk* mutants, which may result from the partially blocked late endosomal fusion to form vacuoles.

TFEB, a key protein regulating lysosomal biogenesis, is also involved in the biogenesis of LROs^45^. We found that deletion of *Dstyk* promoted the phosphorylation of TFEB and repressed TFEB nuclear translocation. Moreover, levels of *tfeb* and several *tfeb* lysosomal target genes were downregulated in *dstyk* mutants. These data suggest that dysregulated TFEB activity may be involved in the pathogenesis of the scoliosis in *dstyk* mutants. Since TFEB activities can be inhibited through its phosphorylation by active mTORC1^43^, and overactivation of mTORC1 results in congenital spinal deformity in mice that can be rescued by mTORC1 inhibitor^50^. We tested whether mTORC1 pathway is involved in the pathogenesis of CS in *dstyk* mutants through its phosphorylation of TFEB. We found that the activity of mTORC1 was increased in *Dstyk*-deficient COS-7 cells and mutant notochord. Treatment with mTORC1 inhibitor Torin1 can promote TFEB nuclear translocation and increase the protein levels of LAMP1 in *Dstyk*-deficient COS-7 cells. Furthermore, Torin1 treatment or *mtor* knockdown with MO can partially rescue the defects in vacuole biogenesis and body curvature in *dstyk* mutants. The detailed molecular mechanisms for the interaction among DSTYK, TFEB and mTOR need to be further investigated. Both starvation or Torin1 treatment inhibit mTORC1, and promote TFEB nuclear translocation, but there’s a difference in nuclear translocation of TFEB upon starvation and Torin1 treatment in our model. We speculate that starvation can only partially suppress mTORC1 and activate TFEB indirectly^51^, while Torin1 is able to fully suppress mTORC1 activity and activate TFEB. In addition, knockdown of *Dstyk* may through an unknown mechanism directly activate mTORC1.

We also explored how the *dstyk* mutants developed into scoliosis at adult stages, according to our data and related references^36,37,52^. We think that *dstyk* mutation leads to scoliosis in zebrafish mainly through its resultant dysregulated biogenesis of notochord vacuoles. *Dstyk* is expressed mainly in notochord cells without observable expression in notochord sheath cells. Since sheath cells are responsible for the notochord sheath formation and mineralization, the absent expression of *dstyk* in sheath cells indicates that the malformation of notochord sheath in *dstyk* mutants is secondary to the dysregulated notochord vacuole biogenesis. Indeed, notochord is a source of developmental signals playing a crucial role in notochord sheath development^31^. Previous studies in zebrafish showed that the shealth formation is closely related to the differentiation of notochord cells^53^. Interestingly, we found the expression of *shh* was decreased in notochord (Fig. 5d). The changed expression of *shh*, a major secreted morphogen in notochord, may affect the development and function of notochord sheath cells, major responsible cells for the sheath development, which may finally lead to disordered notochord sheath segmentation and irregular mineralization order of chordacentra, as well as irregularly sized vertebrae^36,37^ (Fig. 1h, Fig. 7b–e). Nevertheless, only disordered mineralization of chordacentra is not fully responsible for the severe scoliosis^36,37,54^. In addition, the maldeveloped notochord may also mechanically affect sheath development. The pressure exerted by notochord vacuoles on the notochord sheath endows this structure with a characteristic stiffness and mechanical strength^53^. Abnormal formation of vacuoles in *dstyk* mutants results in decreased stiffness and rigidity of notochord sheath that is unable to resist the cranial-to-caudal spinal loads during zebrafish’s swimming. Thus, these physical and mechanical alterations may also be involved in the malformation of notochord sheath including wavy structure with disordered organization and nonuniform thickness of sheath layers (Fig. 6e–h). Since several studies have shown that notochord sheath provides a template for bone mineral deposition and spine patterning, the centra are formed by the direct mineralization of the extracellular notochord sheath and ultimately the elaboration of the vertebral bodies is carried out by the actions of sclerotome-derived osteoblasts on these segmented templates^37,52,55^. Similar study has also shown that disorganization of the extracellular notochord sheath results in congenital vertebral malformations^6^. Therefore, we suggest that the wavy and malformed notochord sheath in *dstyk* mutants may be involved in the chordacentra malformation, and then give rise to defect spine formation. In summary, we speculate that dysregulated notochord vacuole biogenesis and its secondary effect on notochord sheath cells and notochord sheath development together lead to defect spine formation and scoliosis in *dstyk* mutants (Fig. 10a).

We found that *dstyk* mutants had malformed neural and haemal arches similar to the somite clock mutants that have disrupted segmented sclerotome^36,49,56^. We found that the orientation of myofibers was gradually disturbed resulting in irregular and less uniformed arrangement in the *dstyk* mutants (Supplementary Fig. 9), and some somite boundaries in mutants were not as sharp and straight as those in WT (Fig. 7g). Thus, we speculate that the aberrantly compressed and shortened somites may influence the sclerotome and myotome formation at later stage, over time, combined with persistent swim stress, may lead to arch defects. In addition, the notochord is a source of secreted molecules instructing surrounding tissues to acquire specific cell fates^31^. *Shh* is important for somite development including the formation and differentiation of sclerotome and myotome^31^, as the study of Resende et al. revealed that notochord-derived *Shh* temporally controls the presomitic mesoderm segmentation and regulates the pace of the somitogenesis clock^57^. Thus, we speculate that the malformed hemal and neural arches in *dstyk* mutants may be related to the decreased *shh* expression leading to disrupted segmentation clock and sclerotome and myotome formation.

Previous studies reported that patients with a large intragenic deletion of *DSTYK* as the genetic basis for Spastic Paraparesis type 23 (SPG23), an autosomal-recessive disorder characterized by progressive spastic paraplegia and skin pigment abnormalities^58^. In fact, melanosomes in skin and hair melanocytes also belong to LROs, and disordered melanosome biogenesis has been found to result in pigment abnormalities^38^. We here found that DSTYK is involved in both the notochord vacuole biogenesis and lysosome formation. Furthermore, defect biogenesis or function of lysosomes is one of the main mechanisms underlying hereditary spastic paraplegia (HSP)^59^. Two HSP proteins, spastizin (SPG15) and spatacsin (SPG11), are involved in generating new lysosomes via autophagic lysosome reformation^60^, and mutations of the HSP genes spastin (SPG4), strumpellin (SPG8), and REEP1 (SPG31) also affect lysosomal function^59^. We speculate that the pigment phenotypes and spastic paraplegia in SPG23 patients may result from the biogenesis defect of lysosomes and LROs. The defect biogenesis of LROs and lysosomes appear to be the common underlying abnormality for the pathogenesis of scoliosis, pigment abnormalities and spastic paraplegia. These variables but mechanistically related phenotypes from several species further support the essential role of DSTYK in the biogenesis of LROs and lysosomes.

One intriguing phenomena about the role of DSTYK in the development and homeostasis is the variable phenotypes among *Dstyk* deficient zebrafishes, mice and humans. Patients with heterozygous mutations of *DSTYK* were identified as a frequent cause of autosomal-dominant congenital anomalies of the kidney and urinary tract-1 (CAKUT1), and homozygous mutations were identified as SPG23^40,58^, suggesting the essential role of DSTYK in the human development. Li et al. however, reported that mice with ablation of *Dstyk* kinase domain showed no obvious developmental defects^61^. More importantly, the incidence of scoliosis is inconsistent in different species. While we found all *dstyk* mutant zebrafishes have severe scoliosis, among the seven SPG23 families with mutation of *DSTYK* reported, only patients in three families have scoliosis^58^. These studies indicate that *DSTYK* mutation may lead to scoliosis in human, but we currently do not know whether the scoliosis in those patients with *DSTYK* mutation is related to the direct effect of *DSTYK* mutation on spine development or secondary to the spastic paraparesis.

The reasons for the variable phenotypes among zebrafish, mouse *Dstyk* mutants and human patients are not clarified presently. We speculate it may be related to the following reasons. First, there may be differential DSTYK functions among different species during evolution and there are genetic heterogeneities or genetic modifications in different species. Second, the species differences in the structure and development mechanisms of limbs, spine and central nervous system. The roles of notochord in zebrafish, mouse and human development are not exactly the same, although the notochord as a source of secreted growth factors, has similar role for the proper patterning the surrounding tissues in these species. In mammals, the structural role of notochord is relatively weaker, as they do not require a rigid notochord as a stabilizing element for the early embryo^31^. In addition, vertebral bodies are derived from the somite-derived sclerotomes in mammals while the centra mineralization relies on notochord sheath cells and vacuolized notochord in zebrafish^49^. Finally, it has been reported that the *Dstyk* knockout mice still express truncated DSTYK protein, which may still play some role as the WT DSTYK does in development^61^. In addition, many phenotypes observed in patients with *DSTYK* mutations could not observed in *Dstyk* knockout mice.

In summary, in this study we found that mutation of *dstyk* gene leads to CS-like vertebral malformations in zebrafish mainly by causing defects in biogenesis of notochord vacuole through mTORC1 activity dependent repression of TFEB nuclear translocation. Our study demonstrates an important role of DSTYK in notochord vacuole biogenesis, notochord morphogenesis and spine formation through mTORC1/TFEB pathway, which will deepen our understanding of the LRO biogenesis, notochord and spine development.

## Methods

### Zebrafish strains and generation of transgenic lines

Zebrafish (*Danio rerio*) of the AB genetic background were used. The *Tg(osterix:mCherry)*^62^ was from Juhui Qiu (Chongqing University, Chongqing), *Tg(β-actin:ras-GFP)*^*cq76*^ was described before^27^. The *Tg(col2a1a:EGFP)*^*cq77*^ was generated in our group using 1.7 kb col2a1a promoter^28^, and the *Tg(cyb5r2:GFP)*^*cq81*^ was generated using BAC construct^29^. Three heat shock-inducible transgenic lines *Tg(hsp70l:dstyk-GFP)*^*cq78*^, *Tg(hsp70l:DSTYK-GFP)*^*cq79*^ and *Tg(hsp70l:LAMP1-mCherry)*^*cq80*^ were generated by our laboratory. All zebrafish were housed in semi-closed recirculation housing systems (ESEN, China) and were kept at a constant temperature (27–28 °C) on a 14:10 h light:dark photoperiod. All zebrafish lines were raised and maintained according to standard protocols^63^. All in vivo experiments and protocols were approved by Institutional Animal Care and Use Committee of the Research Institute of Surgery, Daping Hospital IACUC protocol SCXK- (Army) 2007-017.

### ENU mutagenesis

ENU (Sigma, USA) mutagenesis was carried out based on previous studies^23,24^. Briefly, the adult male zebrafish were treated with ENU (3.5 mM) for 1 h at weekly intervals and repeated for six times. Three weeks after the ENU treatment, those male zebrafish were outcrossed to AB females to generate F1 families. Then F1 fish were mated to wild-type to generate F2 families. F2 sibling were intercrossed to generate F3 embryos for screening. The mutagenesis efficiency was tested by the F1 death rate (>50%) of the first two times outcrossing. To identify the mutants with spinal deformity, we analyzing the gross morphology and spine formation through living Calcein skeletal staining at different development stages in F3 embryos.

### Genetic mapping and sequencing

Heterozygous *smt* were outcrossed with the polymorphic line SJD to generate a mapping population. Subsequence mapping was performed according to the method from the Zon laboratory^25^. Briefly, bulk segregant linkage analysis was used to map the mutation to a particular chromosome, high-resolution mapping and chromosomal walking can be used to narrow the genetic interval. Directly sequencing was used to detect actual point mutations and deletion mutations. Seven hundred fifty six meiosis recombinants were collected and analysis. The coding region of mutant *dstyk* was amplified with the forward primer: 5′-CTGCCTCGACCTCTTCATCATAC-3′, reverse primer: 5′-CGTCTGGAGCGAACTGAAGATC-3′. Genomic DNA sequencing amplified from the isolated genomic DNA by PCR using the *dstyk* specific primers: sense, 5′-GACCAACTCGGTGGATTACCTG-3′, antisense, 5′-CTGAGGTGGAGAGCACTCTTGT-3′.

### Generated *dstyk* mutants using CRISPR/Cas9 system

*Dstyk* mutants were generated by targeting the 5th exon of *dstyk* with CRISPR/Cas9 technology. The process was performed according to the method from the Xiong laboratory^64^. Briefly, the humanized Cas9 cDNAs with double NLS plasmid and gRNA scaffold plasmid were obtained from Jing-Wei Xiong lab (Peking University, China). Capped Cas9 mRNA was synthesized using mMESSAGE mMACHINE T7 Transcription Kit (Ambion, AM1344), gRNA was synthesized using T7 RNA Polymerase (NEB, M0251S). The cas9 mRNA (300 ng/µl) and gRNA (20–200 ng/µl) were co-injected into 1 or 2 cell stage embryos. Sanger sequencing was used to screen the mutation of injected embryos. The *dstyk* target sequence was 5′-GGAGAGCTTCGTGGGAACAC-3′, and the target region was amplified by PCR using the following primers: forward primer: 5′-CATCATGTCGTCTAACGGCGAG-3′, reverse primer: 5′-TGTTTGAGATGGTTGGACGTGAC-3′.

### In situ hybridization, antibody staining

The template for *dstyk* antisense probe synthesis was amplified with the following primer, forward primer: 5′-GCGAACTGAAGATCTGTGGTTG-3′, reverse primer: 5′-TCAGCTGCCAACACTCCTCATC-3′. Primers for other probes are available from the corresponding authors upon request. PCR products was cloned into the pGEMT-easy vector (Promega). Digoxigenin-labeled probes were generated by in vitro transcription (DIG RNA Labeling Kit, Roche) and ISH was carried out according to the zebrafish book^63^. Briefly, embryos were fixed with 4% paraformaldehyde in PBS at 4 °C overnight, and dehydrated by methanol series and stored at −20 °C overnight. The embryos were then rehydrated into PBT (PBS + 0.1% tween 20) and pre-hybridization for 2–5 h in hybridization solution with 50% formamide at 68.5 °C. Hybridization was performed with 0.5–1 ng/μl antisense probes diluted in hybridization solution overnight at 68.5 °C. Embryos were washed with 2× SSCT buffer and blocked in 1% blocking reagent (Roche, 11096176001) at room temperature for 1 h. The embryos were then incubated with anti-Dig-AP antibody (Roche, 11093274910, 1:5000) at 4 °C overnight. Embryos were rinsed extensively over a total time of 3 hours, then staining with NBT/BCIP solution (Roche, 11681451001, 1:50). The WISH images were captured using a SteREO Discovery 20 microscope (Carl Zeiss). Whole mount antibody staining was performed according to the zebrafish book with slight modifications^63^. Briefly, embryos were fixed with 4% formaldehyde in PBS, and washed with 0.3% Triton X-100 in PBS. The embryos then blocked in the blocking solution (4% BSA, 0.3% Triton X-100 in PBS) at 4 °C for 2–5 h. The primary antibodies of rabbit anti-pS6 (S235/236) (Cell Signaling Technology, 4858, 1:300), rabbit anti-S6 (Cell Signaling Technology, 2217, 1:300), mouse anti-Type II Collagen (Chondrex, 7005, 1:200), rabbit anti-RAB7A (Proteintech, 55469-1-AP, 1:200) were diluted in the blocking solution and incubated at 4 °C overnight. embryos were then washed with 0.3% Triton X-100 in PBS and incubated with secondary antibodies donkey-anti-Rabbit Alexa Fluor 568 (Invitrogen, A10042, 1:500) overnight at 4 °C. After being washed 4–8 times, the samples were imaged using LSM880NLO confocal microscope (Carl Zeiss).

### Morpholino knockdown and mRNA rescue experiments

Splicing block morpholino (MO) targeting the kinase domain of mtor, 5′-GGTTTGACACATTACCCTGAGCATG-3′, at the concentration of 0.4 mM was injected into yolks at the 1-cell stage. For in vitro mRNA synthesis, the coding sequence of zebrafish *dstyk* and human *DSTYK* were amplified and cloned into the pCS2 (+) vector. Capped mRNAs were synthesized using T7 mMessage mMachine Kit (Ambion) according to the manufacturer’s instructions. The mRNA was injected into yolk at the one-cell stage at the dose of 100 pg/embryo. The mRNA rescue experiments were performed three times and more than 200 embryos (about 100 WT embryos and 100 mutant embryos) were used per experiment.

### Edu cell proliferation assay and TUNEL assay

The Edu cell proliferation assay was applied for S-phase labeling according to the manufacturer’s instructions (Click-iT Edu Imaging Kits, Invitrogen). Embryos were injected with 0.2 mM Edu at 36 hpf and incubated at 28.5 °C incubator for 30 min. After being fixed with 4% PFA at 4 °C overnight, embryos were subjected to Edu assay. The TUNEL assay was performed to label apoptosis in the notochord at 36 hpf, using in situ Cell Death Detection kit TMR Red (Roche) as described by the manufacturer.

### Live staining and imaging

Zebrafish in vivo skeletal staining was incubated with 0.2% Calcein (Sigma, C0875) solution (pH 7.0) for 10 min or 0.2% Calcein blue (Sigma, M1255) solution (pH 7.0) for 1 h or 0.05% Alizarin red (Sigma, A5533) for 1 h and then washed with system water three times. Cell internal membranes labeled with vital dye BODIPY TR Methyl Ester (C34556, Invitrogen) as described by the manufacturer, the embryos were stained with 100 μM MED for 1 h, then wash three times with egg water. BODIPY TR Ceramide (D7540, Invitrogen) prominent labeling of the Golgi apparatus with 50 μM for 2 h. LysoTracker Green DND-26 (L7526, Invitrogen) was used to in vivo label acidic notochord vacuoles after 5–6 dpf, 50 μM stained for 1–2 h depending on acidification degree of notochord vacuoles. Zebrafish embryos were anesthetized in tricaine (Sigma) and mounted in 1% LMP agarose. Calcein staining embryos were imaged with a SteREO Discovery 20 microscope (Carl Zeiss). Other live staining embryos were imaged with a LSM880NLO confocal microscope with a ×20 water immersion objective (Carl Zeiss). Images collected every 10 min for time series live cell imaging of notochord development from 20 to 30 hpf.

### Cell culture and transfection

COS-7 and HeLa cell lines were kindly provided by Stem Cell Bank, Chinese Academy of Sciences (Shanghai, China), and cultured in Dulbecco’s modified Eagle’s medium (DMEM) supplemented with 10% fetal bovine serum (FBS). All the cells were maintained at 37 °C in 5% CO_2_-containing atmosphere. The medium and serum were purchased from Hyclone. For *Dstyk* siRNA knockdown, cells were transfected with 100 pmol RNA oligonucleotides using Lipofectamine RNAiMAX transfection reagent (Invitrogen). *DSTYK-V5* plasmids were transfected with Lipofectamine 3000 (Invitrogen).

### Cell immunofluorescence staining

For immunofluorescence assay, cells were grown in coverslips and transfected with siRNA or indicated plasmids. After 36–48 h transfection, cells were washed with PBS and fixed with 4% formaldehyde. The embryos then blocked with blocking solution (3% BSA, 0.3% Triton X-100 in PBS) for 30 min, and incubated with primary antibodies, including rabbit anti-mTOR (Cell Signaling Technology, 2983, 1:300), mouse anti-DSTYK (Santa Cruz, sc-374487, 1:100), mouse anti-LAMP1 (Santa Cruz, sc-20011, 1:100), rabbit anti-V5 (Proteintech, 14440-1-AP, 1:200), rabbit anti-RAB7A (Proteintech, 55469-1-AP, 1:200), rabbit anti-DSTYK (Proteintech, 20102-1-AP, 1:200), rabbit anti-TFEB (Proteintech, 13372-1-AP, 1:200), mouse anti-V5 (Abcam, ab27671, 1:300), overnight at 4 °C. Alexa Fluor 488 donkey anti-rabbit IgG (Invitrogen, A21206, 1:500), Alexa Fluor 568 donkey anti-rabbit IgG (Invitrogen, A10042, 1:500) and Alexa Fluor 594 donkey anti-mouse IgG (Invitrogen, A21203, 1:500) were used as a secondary antibody. Immunofluorescence staining was imaged with LSM880NLO confocal microscope with a ×63 oil objective (Carl Zeiss).

### Western blot analysis

Zebrafish embryos at 48 hpf and cultured COS-7 cells were lysed using RIPA lysis buffer containing protease inhibitors (Roche). Equal amount of protein samples was resolved on a 10% or 12% SDS-PAGE gel and transferred onto a PVDF membrane (Millipore). Then samples were probed with the primary antibodies, including rabbit anti-S6K (Cell Signaling Technology, 2708, 1:1000), rabbit anti-p-S6K (T389) (Cell Signaling Technology, 9205, 1:1000), mouse anti-DSTYK (Santa Cruz, sc-374487, 1:500), mouse anti-LAMP1 (Santa Cruz, sc-20011, 1:500), rabbit anti-TFEB (Proteintech, 13372-1-AP, 1:1000), rabbit anti-LAMP3 (Proteintech, 12632-1-AP, 1:1000), mouse anti-actin (Sigma, A5316, 1:5000), overnight at 4 °C. Secondary antibodies goat anti-rabbit IgG (ZSGB-BIO, ZB-2301, 1:5000), goat anti-mouse IgG (ZSGB-BIO, ZB-2305, 1:5000) was used, as a secondary antibody, followed by detection with Pierce™ ECL Western Blotting Substrate (Thermo Scientific, 32106). Uncropped images of western blotting and gel are shown in the Source Data file.

### Transmission electron microscopy

For TEM, zebrafish embryos at 48 hpf were fixed with 2.5% glutaraldehyde and post-fixed with 1.0% osmium tetroxide, dehydrated in graded concentration of acetone, and embedded in epon. Ultrathin sections (70 nm) were cut using a Leica Ultracut UCT ultramicrotome and subsequently stained with lead citrate and uranyl acetate. Specimens were visualized using an electron microscope (JEM-1400Plus, Japan).

### X -ray and micro-computed tomography

Three-month adult zebrafish were harvested, stored in 75% ethanol at 4 °C, and were subjected to high-resolution X-rays examination using Faxitron MX20. The zebrafish were scanned with micro-CT (viva CT-40, Scanco Medical AG, Switzerland). Image acquisition was performed with the condition of 45 kV and 177 μA in high-resolution scans (10.5 µm voxel resolution). Two-dimensional images were used to generate three-dimensional reconstructions. Every measurement used the same filtering and segmentation values. The images were analyzed by micro-CT Evolution Program V6.5 software.

### Heat shock

Embryo heat shocking was performed by rapidly immersing 72 hpf embryos into 39 °C egg water for 30 min to induce hsp70l expression, followed by gradual recovery to 28.5 °C. Embryos were imaged with LSM880NLO confocal microscope at about 78 hpf.

### Drug treatment

Torin1 was purchased from Selleck chemicals. Torin1 dissolved with DMSO to get a stock solution of 1 mM. For cell treatment, Torin1 were used in 1 μM for 3 h. For zebrafish treatment, a final concentration of 500 nM Torin1 were treated during notochord development from 20 to 30 hpf. The embryos were labeled using BODIPY TR methyl ester, and notochord vacuole size measurements were conducted using Image J.

### Statistics

All numeric data are presented as mean ± SD. Error bars indicate SD. Differences between two groups were evaluated using Unpaired Student’s *t* test, and ANOVA was used for comparisons of multiple groups. When significant levels (*P* < 0.05) were achieved, Tukey’s Post Hoc test was performed. All statistical analyses were performed using GraphPad PRISM 7.0 software, and *P* < 0.05 was considered statistically significant.

### Reporting summary

Further information on research design is available in the Nature Research Reporting Summary linked to this article.

## Supplementary information

Supplementary Information

Reporting Summary

Description of Additional Supplementary Files

Supplementary Movie 1

Supplementary Movie 2

## Data Availability

The raw data that support the findings of this study are available from the corresponding author upon reasonable request. A reporting summary for this Article is available as a Supplementary Information file. The source data underlying Figs. 1b, f, 2e, 4c, d, 7f, 8g–i and 9a–d, f, h, l and Supplementary Figs 1a–d, 2b, d, 3c, d and 7a, e, f are provided as a Source Data file.

## Source Data

Source Data

## References

[1] Weiss HR, Moramarco M (2016). Congenital scoliosis (Mini-review). Curr. Pediatr. Rev..

[2] Sparrow DB (2012). A mechanism for gene-environment interaction in the etiology of congenital scoliosis. Cell.

[3] Giampietro PF (2009). Progress in the understanding of the genetic etiology of vertebral segmentation disorders in humans. Ann. N. Y. Acad. Sci..

[4] Giampietro PF (2013). Clinical, genetic and environmental factors associated with congenital vertebral malformations. Mol. Syndromol..

[5] Beauregard-Lacroix Eliane, Tardif Jessica, Camurri Maria Vittoria, Lemyre Emmanuelle, Barchi Soraya, Parent Stefan, Campeau Philippe M. (2017). Retrospective Analysis of Congenital Scoliosis. SPINE.

[6] Gray RS (2014). Loss of col8a1a function during zebrafish embryogenesis results in congenital vertebral malformations. Dev. Biol..

[7] Christiansen HE, Lang MR, Pace JM, Parichy DM (2009). Critical early roles for col27a1a and col27a1b in zebrafish notochord morphogenesis, vertebral mineralization and post-embryonic axial growth. PLoS ONE.

[8] Bulman MP (2000). Mutations in the human delta homologue, DLL3, cause axial skeletal defects in spondylocostal dysostosis. Nat. Genet..

[9] Sparrow DB (2013). Mutation of HES7 in a large extended family with spondylocostal dysostosis and dextrocardia with situs inversus. Am. J. Med. Genet. Part A.

[10] Whittock NV (2004). Mutated MESP2 causes spondylocostal dysostosis in humans. Am. J. Hum. Genet..

[11] Wu N (2015). TBX6 null variants and a common hypomorphic allele in congenital scoliosis. N. Engl. J. Med..

[12] Janssen MMA, de Wilde RF, Kouwenhoven J-WM, Castelein RM (2011). Experimental animal models in scoliosis research: a review of the literature. Spine J..

[13] Grimes DT (2016). Zebrafish models of idiopathic scoliosis link cerebrospinal fluid flow defects to spine curvature. Science.

[14] Gorman KF, Breden F (2007). Teleosts as models for human vertebral stability and deformity. Comp. Biochem. Physiol. Part C: Toxicol. Pharmacol..

[15] Boswell CW, Ciruna B (2017). Understanding idiopathic scoliosis: a new zebrafish school of thought. Trends Genet..

[16] Kou I (2013). Genetic variants in GPR126 are associated with adolescent idiopathic scoliosis. Nat. Genet..

[17] Guo L (2016). Functional investigation of a non-coding variant associated with adolescent idiopathic scoliosis in zebrafish: elevated expression of the ladybird homeobox gene causes body axis deformation. PLoS Genet..

[18] Hayes M (2014). ptk7 mutant zebrafish models of congenital and idiopathic scoliosis implicate dysregulated Wnt signalling in disease. Nat. Commun..

[19] Buchan JG (2014). Kinesin family member 6 (kif6) is necessary for spine development in zebrafish. Dev. Dyn..

[20] Patten SA (2015). Functional variants of POC5 identified in patients with idiopathic scoliosis. J. Clin. Investig..

[21] Gao W (2017). Rare coding variants in MAPK7 predispose to adolescent idiopathic scoliosis. Hum. Mutat..

[22] Sharma S (2015). A PAX1 enhancer locus is associated with susceptibility to idiopathic scoliosis in females. Nat. Commun..

[23] Mullins MC, Hammerschmidt M, Haffter P, Nusslein-Volhard C (1994). Large-scale mutagenesis in the zebrafish: in search of genes controlling development in a vertebrate. Curr. Biol..

[24] Solnica-Krezel L, Schier AF, Driever W (1994). Efficient recovery of ENU-induced mutations from the zebrafish germline. Genetics.

[25] Zhou Y, Zon LI (2011). The zon laboratory guide to positional cloning in zebrafish. Methods Cell Biol..

[26] Ellis K, Bagwell J, Bagnat M (2013). Notochord vacuoles are lysosome-related organelles that function in axis and spine morphogenesis. J. Cell Biol..

[27] Lu H, Ma J, Yang Y, Shi W, Luo L (2013). EpCAM is an endoderm-specific Wnt derepressor that licenses hepatic development. Dev. Cell.

[28] Dale RM, Topczewski J (2011). Identification of an evolutionarily conserved regulatory element of the zebrafish col2a1a gene. Dev. Biol..

[29] Yamamoto M (2010). Mib-Jag1-Notch signalling regulates patterning and structural roles of the notochord by controlling cell-fate decisions. Development.

[30] Cooper MS (2005). Visualizing morphogenesis in transgenic zebrafish embryos using BODIPY TR methyl ester dye as a vital counterstain for GFP. Dev. Dyn..

[31] Corallo D, Trapani V, Bonaldo P (2015). The notochord: structure and functions. Cell. Mol. life Sci..

[32] Coutinho P (2004). Differential requirements for COPI transport during vertebrate early development. Dev. Cell.

[33] Cerda J, Grund C, Franke WW, Brand M (2002). Molecular characterization of Calymmin, a novel notochord sheath-associated extracellular matrix protein in the zebrafish embryo. Dev. Dyn..

[34] Scott A, Stemple DL (2005). Zebrafish notochordal basement membrane: signaling and structure. Curr. Top. Dev. Biol..

[35] Corallo D (2013). Emilin3 is required for notochord sheath integrity and interacts with Scube2 to regulate notochord-derived Hedgehog signals. Development.

[36] Lleras Forero, L. et al. Segmentation of the zebrafish axial skeleton relies on notochord sheath cells and not on the segmentation clock. *eLife* **7**, e33843 (2018). 10.7554/eLife.33843 PMC5962341 29624170

[37] Wopat S (2018). Spine patterning is guided by segmentation of the notochord sheath. Cell Rep..

[38] Huizing M, Helip-Wooley A, Westbroek W, Gunay-Aygun M, Gahl WA (2008). Disorders of lysosome-related organelle biogenesis: clinical and molecular genetics. Annu. Rev. Genomics Hum. Genet..

[39] Peng J (2006). Dusty protein kinases: primary structure, gene evolution, tissue specific expression and unique features of the catalytic domain. Biochimica et. Biophysica Acta.

[40] Sanna-Cherchi S (2013). Mutations in DSTYK and dominant urinary tract malformations. N. Engl. J. Med..

[41] Settembre C (2011). TFEB links autophagy to lysosomal biogenesis. Science.

[42] Sardiello M (2009). A gene network regulating lysosomal biogenesis and function. Science.

[43] Roczniak-Ferguson A (2012). The transcription factor TFEB links mTORC1 signaling to transcriptional control of lysosome homeostasis. Sci. Signal..

[44] Settembre C (2012). A lysosome-to-nucleus signalling mechanism senses and regulates the lysosome via mTOR and TFEB. EMBO J..

[45] Settembre C, Fraldi A, Medina DL, Ballabio A (2013). Signals from the lysosome: a control centre for cellular clearance and energy metabolism. Nat. Rev. Mol. Cell Biol..

[46] Sancak Y (2008). The Rag GTPases bind raptor and mediate amino acid signaling to mTORC1. Science.

[47] Eskelinen EL (2006). Roles of LAMP-1 and LAMP-2 in lysosome biogenesis and autophagy. Mol. Asp. Med..

[48] Zha J (2004). RIP5 is a RIP-homologous inducer of cell death. Biochem. Biophys. Res. Commun..

[49] Fleming A, Keynes R, Tannahill D (2004). A central role for the notochord in vertebral patterning. Development.

[50] Yang C (2017). Chondrocyte-specific knockout of TSC-1 leads to congenital spinal deformity in mice. BioMed. Res. Int..

[51] Marat AL (2017). mTORC1 activity repression by late endosomal phosphatidylinositol 3,4-bisphosphate. Science.

[52] Huitema LF (2012). Entpd5 is essential for skeletal mineralization and regulates phosphate homeostasis in zebrafish. Proc. Natl Acad. Sci. USA.

[53] Trapani V, Bonaldo P, Corallo D (2017). Role of the ECM in notochord formation, function and disease. J. Cell Sci..

[54] Lopez-Baez, J. C. et al. Wilms Tumor 1b defines a wound-specific sheath cell subpopulation associated with notochord repair. *eLife* **7**, e30657 (2018). 10.7554/eLife.30657 PMC5811212 29405914

[55] Wang S (2013). Mineralization of the vertebral bodies in Atlantic salmon (Salmo salar L.) is initiated segmentally in the form of hydroxyapatite crystal accretions in the notochord sheath. J. Anat..

[56] van Eeden FJ (1996). Mutations affecting somite formation and patterning in the zebrafish, Danio rerio. Development.

[57] Resende TP (2010). Sonic hedgehog in temporal control of somite formation. Proc. Natl Acad. Sci. USA.

[58] Lee JYW (2017). Large intragenic deletion in DSTYK underlies autosomal-recessive complicated spastic paraparesis, SPG23. Am. J. Hum. Genet..

[59] Sharma J, Ronza AD, Lotfi P, Sardiello M (2018). Lysosomes and brain health. Annu. Rev. Neurosci..

[60] Chang J, Lee S, Blackstone C (2014). Spastic paraplegia proteins spastizin and spatacsin mediate autophagic lysosome reformation. J. Clin. Investig..

[61] Li K (2014). DSTYK kinase domain ablation impaired the mice capabilities of learning and memory in water maze test. Int. J. Clin. Exp. Pathol..

[62] Singh SP, Holdway JE, Poss KD (2012). Regeneration of amputated zebrafish fin rays from de novo osteoblasts. Dev. Cell.

[63] Westerfield M. *The Zebrafish Book: A Guide for the Laboratory Use of Zebrafish (Danio rerio)*. (M. Westerfield, 2007).

[64] Chang N (2013). Genome editing with RNA-guided Cas9 nuclease in zebrafish embryos. Cell Res..

