# Supplementary material for: Dstyk mutation leads to congenital scoliosis-like vertebral malformations in zebrafish via dysregulated mTORC1/TFEB pathway
Source: Nat Commun. 2020 Jan 24;11:479. doi: 10.1038/s41467-019-14169-z (PMC6981171; doi:10.1038/s41467-019-14169-z)
Supplement: Supplementary file 1 — Supplementary Information [file 41467_2019_14169_MOESM1_ESM.pdf]

Supplementary Information

Dstyk mutation leads to congenital scoliosis-like vertebral malformations in zebrafish via dysregulated mTORC1/TFEB pathway

Sun et al.

Supplementary Figure 1

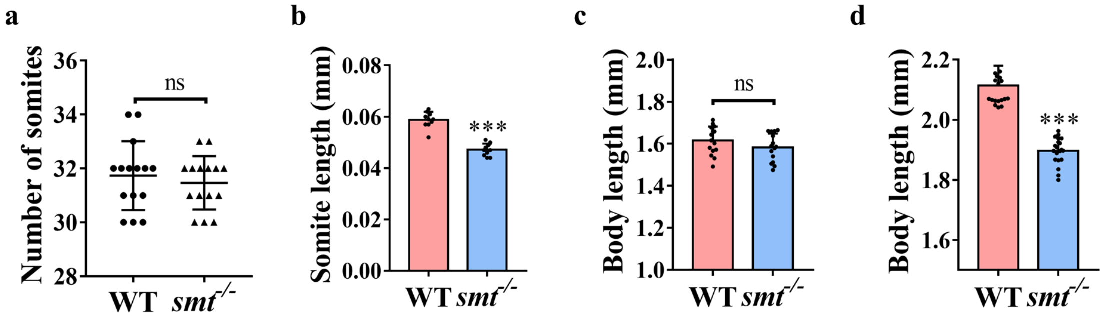

Supplementary Figure 1. Quantification of somite number, somite lengths and body lengths. (**a**) Quantification of average number of somites in WT and *smt* mutants at 4 dpf showed no significant (ns) difference. n=15. (**b**) Quantification of somite lengths in WT and *smt* mutant at 28 hpf. n=10. ***$p < 0.001$. (**c, d**) Quantification of body lengths in WT and *smt* mutants at 20 hpf (**c**) (n=10) and at 28 hpf (**d**) n=20. ***$p < 0.001$. p values in **a-d** determined by unpaired two-tailed Student's t-test. Data are presented as mean ± SD. Source data are provided as a Source Data file.

Supplementary Figure 2

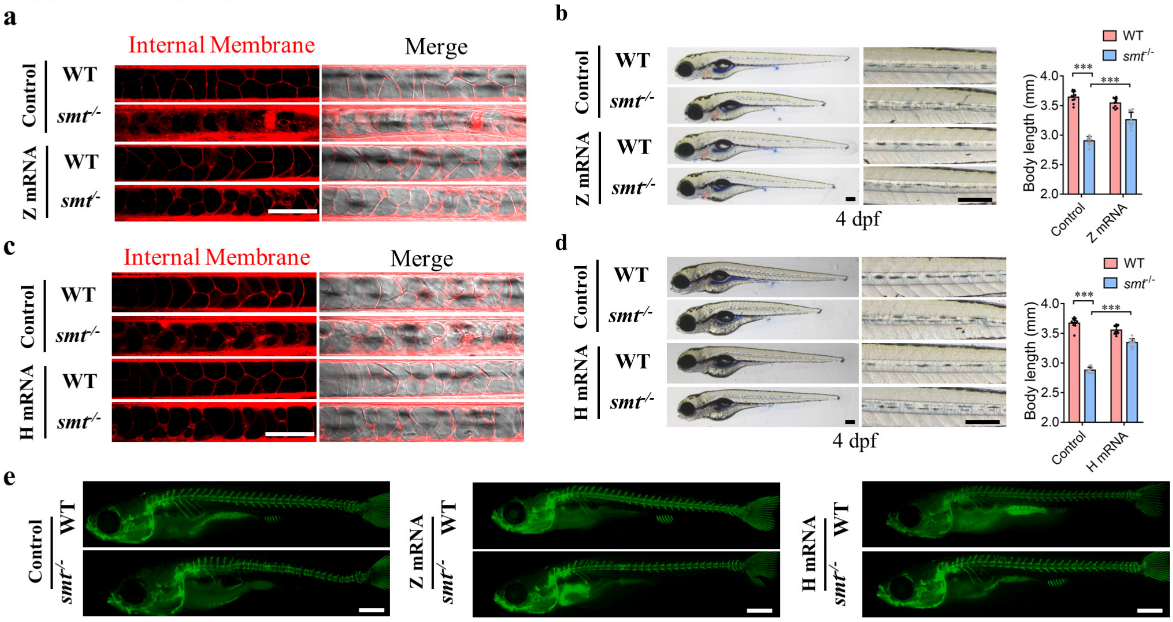

Supplementary Figure 2. Zebrafish *dstyk* mRNA and human *DSTYK* mRNA can rescue the phenotypes of *smt* mutants. (**a-d**) WT and *smt* mutants were injected with 100 pg zebrafish *dstyk* mRNA (Z mRNA) (**a**, **b**) or human *DSTYK* mRNA (H mRNA) (**c**, **d**) at one-cell stage. Confocal images showed vacuole inflation after MED staining at 4 dpf (**a**, **c**). Bright-field images show that gross phenotype was rescued after mRNA injection at 4 dpf (**b**, **d**). Magnified images in the middle column. Quantification of body lengths in the right column (n=10). ***$p < 0.001$. p values in **b** and **d** determined by a two-way ANOVA test. (**e**) Calcein staining images of about 30 dpf WT (top) and *smt* mutants (bottom) after being injected with zebrafish *dstyk* mRNA (middle), human *DSTYK* mRNA (right) or control (left). Data are presented as mean ± SD. Scale bar, 100 µm in **a**, **c**, 200 µm in **b**, **d, 1** mm in **e**. Source data are provided as a Source Data file.

Supplementary Figure 3

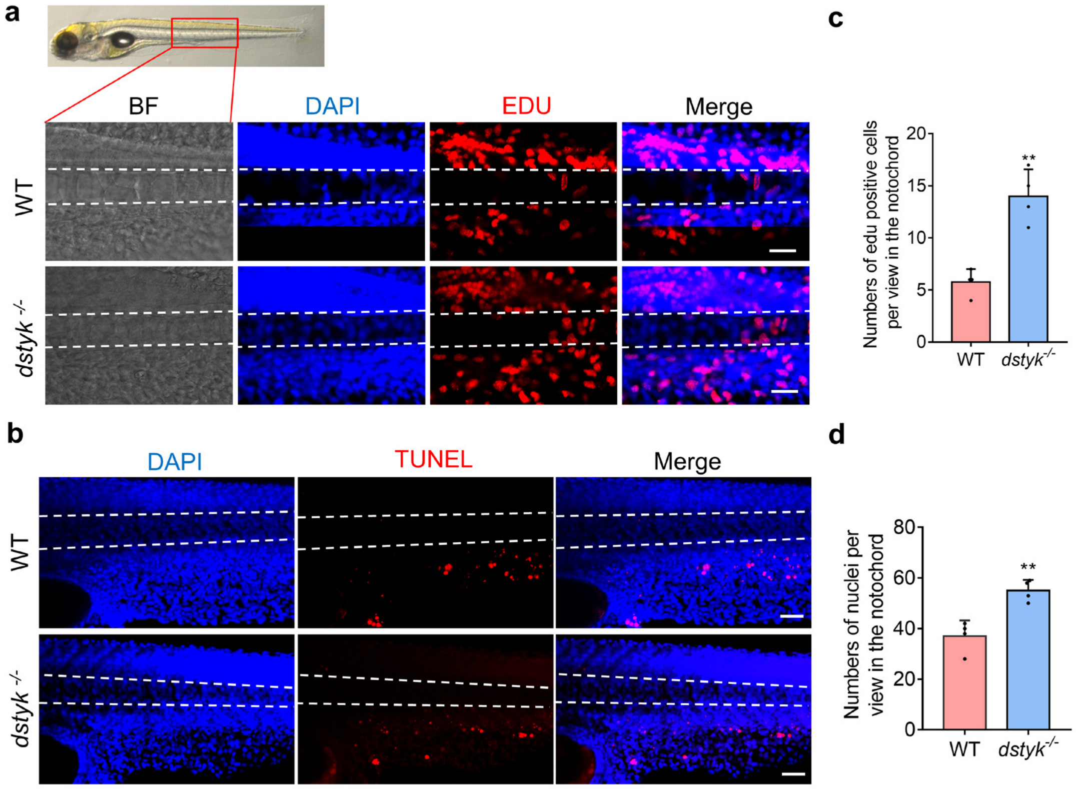

Supplementary Figure 3. Proliferation and apoptosis of cells in the notochord. (**a**) Edu assay showed the cell proliferation in the notochord of WT and the mutant at 36 hpf. Scale bar, 20 µm. (**b**) TUNEL assay showed no apoptosis in the notochord of WT and *dstyk* mutant at 36 hpf. Scale bar, 50 µm. Dashed lines indicate the notochord. (**c**) Quantification of numbers of Edu positive cells in each microscopic field in (**a**). n=4. (**d**) Quantification of nuclei numbers in each microscopic field in (**a**). n=4. ** $p < 0.01$. p values determined by unpaired two-tailed Student's t-test. Data are presented as mean ± SD. Source data are provided as a Source Data file.

Supplementary Figure 4

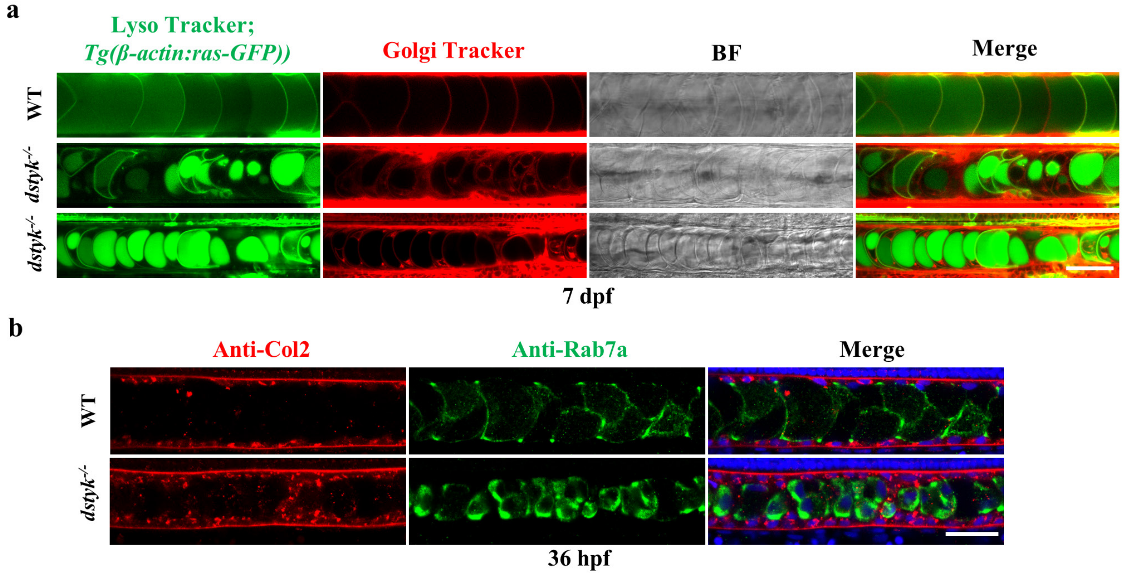

Supplementary Figure 4. Late endosomal trafficking to form vacuoles was partially blocked in the *dstyk* mutants. (**a**) Live confocal images of notochord for WT and *dstyk* mutants dyed with BODIPY TR Ceramide and LysoTracker Green at 7 dpf in the background of *Tg(β-actin:ras-GFP)*(Some cell membranes were labeled with EGFP). Note that the defect of vacuoles biogenesis in the middle panel was severe than the bottom panel, and the size of round cystic structure is bigger in the middle panel. (**b**) Confocal images of double immunofluorescent staining of Rab7a and Col2 of WT and *dstyk* mutants at 36 hpf. Scale bar, 50 µm in **a**, **b**.

Supplementary Figure 5

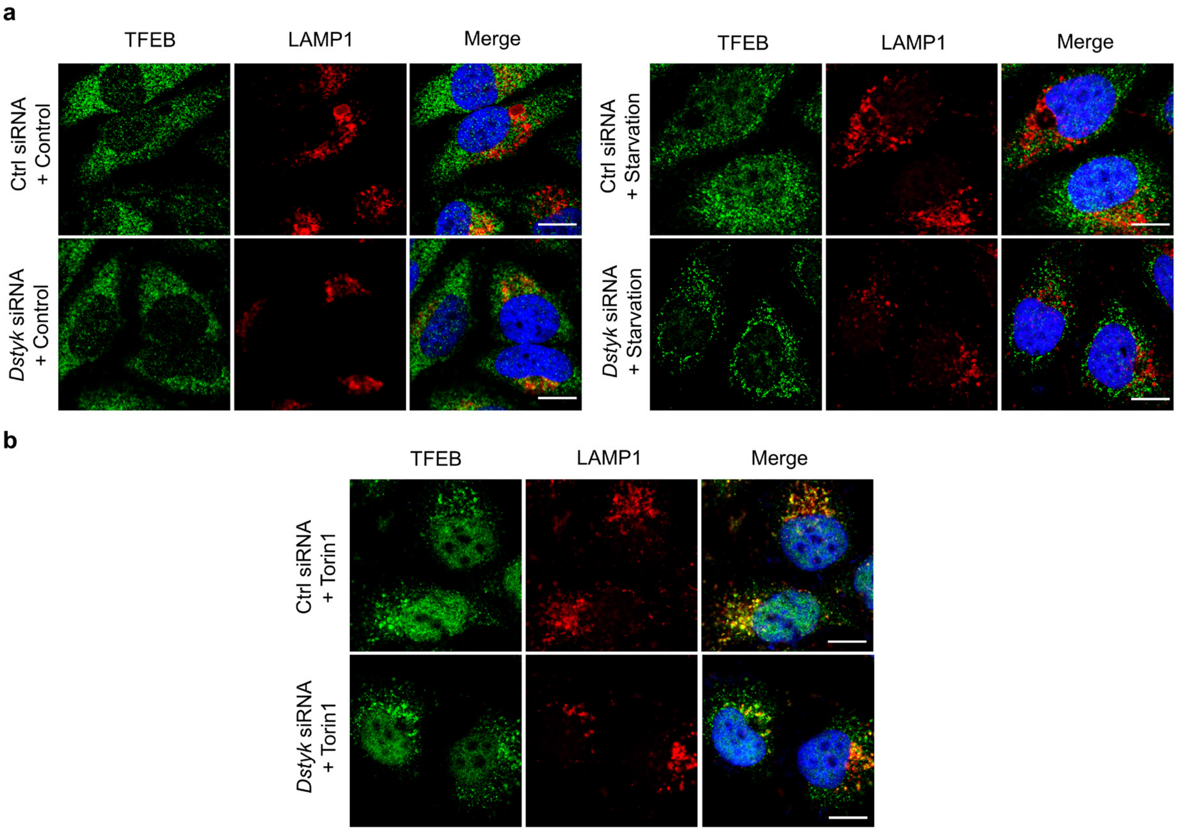

Supplementary Figure 5. *DSTYK* knockdown represses TFEB nuclear translocation and inhibit lysosome biogenesis in HeLa cells. (**a**) HeLa cells transfected with control siRNA or *Dstyk* siRNA, without starvation (left panel) or starved for 2 h (right panel) and analyzed by immunofluorescence for TFEB and LAMP1. (**b**) HeLa cells transfected with control siRNA or *Dstyk* siRNA and treated with Torin1 (1 μM) for 3 h and analyzed by immunofluorescence for TFEB and LAMP1. Scale bars, 10 μm.

Supplementary Figure 6

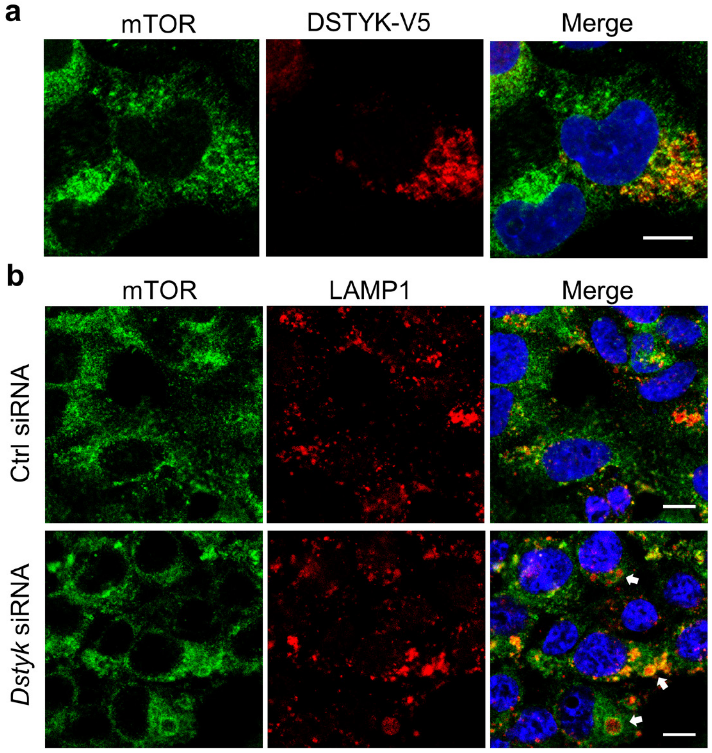

Supplementary Figure 6. DSTYK was co-localized with mTOR and DSTYK knock down promote mTOR lysosomal localization. (**a**) COS-7 cells transfected with DSTYK-V5 and double immunofluorescent staining of V5 (red) and mTOR (green). Note that DSTYK-V5 has much co-localization with mTOR. (**b**) COS-7 cells transfected with control siRNA or *Dstyk* siRNA and starved for 2 h and analyzed by immunofluorescence for mTOR and LAMP1. White arrows indicate that some mTOR moved to small vesicular structures and co-localized with LAMP1. Scale bars, 10 μm.

Supplementary Figure 7

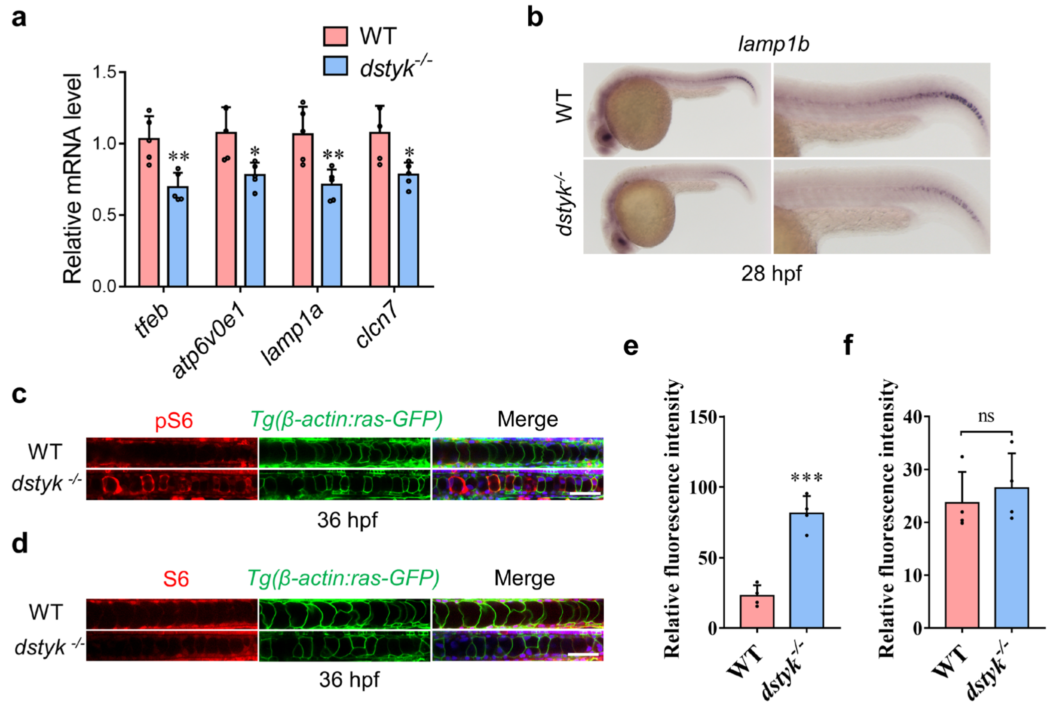

Supplementary Figure 7. *Dstyk* knock out downregulate TFEB target genes and activate mTORC1 in the notochord. (**a**) Expression analysis of WT and *dstyk* mutants showing significant downregulation of *tfeb* and *tfeb* target genes at 36 hpf. Gene expression was normalized relative to the housekeeping gene, GAPDH. *n*=5 independent experiments. * $p < 0.05$, ** $p < 0.01$. (**b**) *In situ* hybridization showed the expression of *tfeb* target gene, *lamp1b* at 28 hpf of WT and *dstyk* mutants. Magnified images in the right column. (**c, d**) Confocal images of immunofluorescent staining of WT and *dstyk* mutant for pS6 (**c**) and S6 (**d**) at 36 hpf in the background of *Tg(β-actin:ras-GFP)*. (**e, f**) Graph depicting the quantification of fluorescence intensity of notochord in (**c**) and (**d**)**.** *n*=4 independent experiments. *** $p < 0.001$. *p* values in **a**, **e** and **f** determined by unpaired two-tailed Student's *t*-test. Data are presented as mean ± SD. Source data are provided as a Source Data file.

Supplementary Figure 8

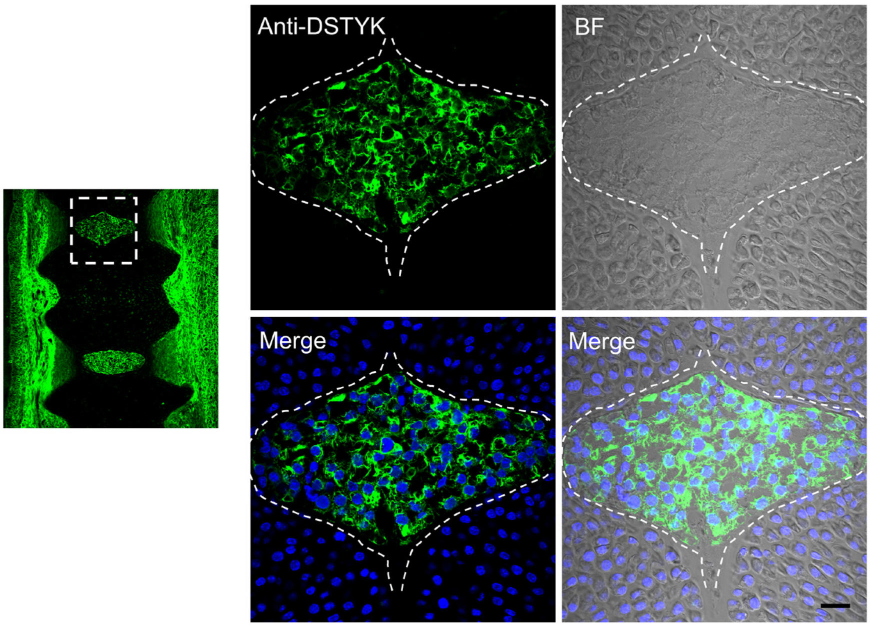

Supplementary Figure 8. DSTYK is expressed in the mouse notochord. Confocal images of immunofluorescent staining for DSTYK in the mouse notochord at embryo 16.5 day. Longitudinal sections of the mouse tail vertebra. Boxed regions in the left panel are magnified in the right panel. Dashed lines indicate the notochord. Scale bars, 20 μm.

Supplementary Figure 9

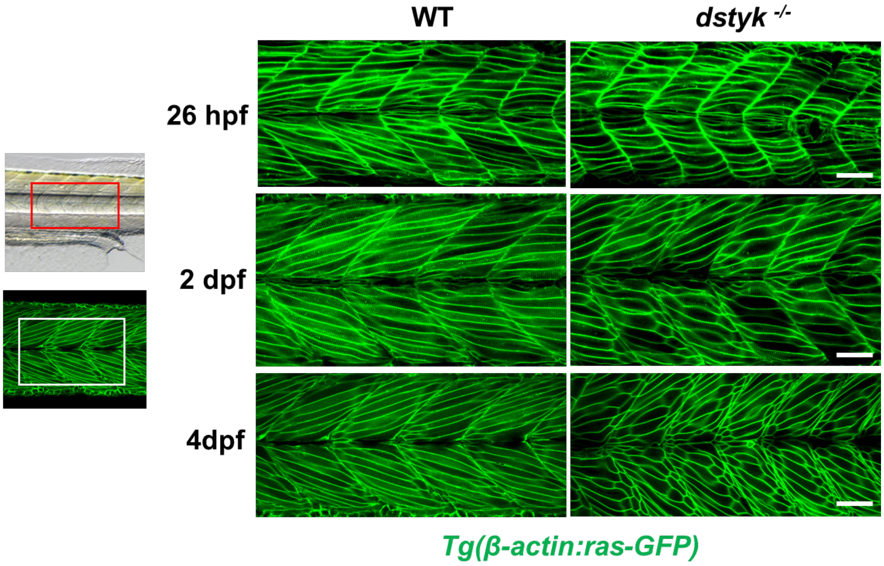

Supplementary Figure 9. Live confocal images showing the myofiber arrangement of WT and *dstyk* mutant in *Tg(β-actin:ras-GFP)* transgenic background from 26 hpf to 4 dpf. All cell membranes were labeled by GFP. Scale bars, 50 µm.
