# Supplementary material for: Dstyk mutation leads to congenital scoliosis-like vertebral malformations in zebrafish via dysregulated mTORC1/TFEB pathway
Source: Nat Commun. 2020 Jan 24;11:479. doi: 10.1038/s41467-019-14169-z (PMC6981171; doi:10.1038/s41467-019-14169-z)
Supplement: Supplementary file 2 — Reporting Summary [file 41467_2019_14169_MOESM2_ESM.pdf]

nature research

Corresponding author(s): Yangli Xie;Lingfei Luo;Lin Chen

Last updated by author(s): Dec 3, 2019

# Reporting Summary

Nature Research wishes to improve the reproducibility of the work that we publish. This form provides structure for consistency and transparency in reporting. For further information on Nature Research policies, see Authors & Referees and the Editorial Policy Checklist.

## Statistics

For all statistical analyses, confirm that the following items are present in the figure legend, table legend, main text, or Methods section.

| n/a | Confirmed | |
|---|---|---|
| ☐ | ☒ | The exact sample size (*n*) for each experimental group/condition, given as a discrete number and unit of measurement |
| ☐ | ☒ | A statement on whether measurements were taken from distinct samples or whether the same sample was measured repeatedly |
| ☐ | ☒ | The statistical test(s) used AND whether they are one- or two-sided<br>*Only common tests should be described solely by name; describe more complex techniques in the Methods section.* |
| ☒ | ☐ | A description of all covariates tested |
| ☒ | ☐ | A description of any assumptions or corrections, such as tests of normality and adjustment for multiple comparisons |
| ☐ | ☒ | A full description of the statistical parameters including central tendency (e.g. means) or other basic estimates (e.g. regression coefficient) AND variation (e.g. standard deviation) or associated estimates of uncertainty (e.g. confidence intervals) |
| ☐ | ☒ | For null hypothesis testing, the test statistic (e.g. *F*, *t*, *r*) with confidence intervals, effect sizes, degrees of freedom and *P* value noted<br>*Give P values as exact values whenever suitable.* |
| ☒ | ☐ | For Bayesian analysis, information on the choice of priors and Markov chain Monte Carlo settings |
| ☒ | ☐ | For hierarchical and complex designs, identification of the appropriate level for tests and full reporting of outcomes |
| ☒ | ☐ | Estimates of effect sizes (e.g. Cohen's *d*, Pearson's *r*), indicating how they were calculated |

*Our web collection on statistics for biologists contains articles on many of the points above.*

## Software and code

Policy information about availability of computer code

| | |
|---|---|
| Data collection | ZEISS ZEN Imaging Software was used for confocal laser scanning microscopy. |
| Data analysis | GraphPad Prism 7.0 (GraphPad Software, Inc.) was used to present data in graphs and for the statistical analyses of the data.<br>ImageJ V1.51 was used to length and size measurements. |

For manuscripts utilizing custom algorithms or software that are central to the research but not yet described in published literature, software must be made available to editors/reviewers. We strongly encourage code deposition in a community repository (e.g. GitHub). See the Nature Research guidelines for submitting code & software for further information.

## Data

Policy information about availability of data

All manuscripts must include a data availability statement. This statement should provide the following information, where applicable:

- Accession codes, unique identifiers, or web links for publicly available datasets
- A list of figures that have associated raw data
- A description of any restrictions on data availability

The raw data that support the findings of this study are available from the corresponding author upon reasonable request.

# Field-specific reporting

Please select the one below that is the best fit for your research. If you are not sure, read the appropriate sections before making your selection.

☒ Life sciences ☐ Behavioural & social sciences ☐ Ecological, evolutionary & environmental sciences

# Life sciences study design

All studies must disclose on these points even when the disclosure is negative.

| | |
|---|---|
| Sample size | It is generally considered that a sample size containing at least 3 biological replicates can provide adequate statistical power in biochemical analysis. We have described the exact sample size for each experiment in our manuscript. |
| Data exclusions | No data captured was excluded from the subsequent analyses. |
| Replication | The exact number of replication for all experiments was described in figure legends and our attempts at replication were successful. |
| Randomization | Samples and animals were allocated randomly. |
| Blinding | The experimenters were blinded to the animal genotype, grouping information and data analysis. |

# Reporting for specific materials, systems and methods

We require information from authors about some types of materials, experimental systems and methods used in many studies. Here, indicate whether each material, system or method listed is relevant to your study. If you are not sure if a list item applies to your research, read the appropriate section before selecting a response.

## Materials & experimental systems

| n/a | Involved in the study | |
|---|---|---|
| ☐ | ☒ | Antibodies |
| ☐ | ☒ | Eukaryotic cell lines |
| ☒ | ☐ | Palaeontology |
| ☐ | ☒ | Animals and other organisms |
| ☒ | ☐ | Human research participants |
| ☒ | ☐ | Clinical data |

## Methods

| n/a | Involved in the study | |
|---|---|---|
| ☒ | ☐ | ChIP-seq |
| ☒ | ☐ | Flow cytometry |
| ☒ | ☐ | MRI-based neuroimaging |

## Antibodies

| | |
|---|---|
| Antibodies used | The following antibodies were used for immunoblotting or immunostaining:abbit anti-S6K (2708), rabbit anti-p-S6K (T389) (9205), rabbit anti-mTOR (2983), rabbit anti-pS6 (S235/236) (4858) and rabbit anti-S6 (2217) were from Cell Signaling Technology, mouse anti-DSTYK (sc-374487), mouse anti-LAMP1 (sc-20011) were from Santa Cruz Biotechnology, rabbit anti-V5 (14440-1-AP), rabbit anti-RAB7A (55469-1-AP), rabbit anti-DSTYK (20102-1-AP), rabbit anti-TFEB (13372-1-AP) and rabbit anti-LAMP3 (12632-1-AP) were from Proteintech, mouse anti-V5 (ab27671) was from Abcam, mouse anti-actin (AC-74, A5316) was from Sigma. Alexa Fluor 488 donkey anti-rabbit IgG (A21206), Alexa Fluor 568 donkey anti-rabbit IgG (A10042) and Alexa Fluor 594 donkey anti-mouse IgG (A21203) were from Invitrogen. |
| Validation | All antibodies are from commercial sources and their validation data are available on the manufacturer's website. The appropriate dilution for all the antibodies was determined through preliminary experiments. |

## Eukaryotic cell lines

Policy information about cell lines

| | |
|---|---|
| Cell line source(s) | The COS-7 and HeLa cell lines were purchased from the Stem Cell Bank, Chinese Academy of Sciences (Shanghai, China). |
| Authentication | The COS-7 and HeLa cell lines were verified by short tandem-repeat DNA profiling before the study. |
| Mycoplasma contamination | All the cell lines had been tested and confirmed negative for mycoplasma contamination. |
| Commonly misidentified lines (See ICLAC register) | No commonly misidentified cells were used in this study. |

## Animals and other organisms

Policy information about studies involving animals; ARRIVE guidelines recommended for reporting animal research

| | |
|---|---|
| Laboratory animals | The detailed information about  zebrafish strains and generation of transgenic lines used in this study has been provided in our METHODS section. The development stage in zebrafish experiments are included in the manuscript. All zebrafish were housed in semi-closed recirculation housing systems (ESEN, China) and were kept at a constant temperature (27-28°C) on a 14:10 hour |

| | |
|---|---|
| | light:dark photoperiod. All in vivo experiments and protocols were approved by Institutional Animal Care and Use Committee of the Research Institute of Surgery, Daping Hospital. |
| Wild animals | No wild animals involved in this study. |
| Field-collected samples | The study did not involve samples collected from the field. |
| Ethics oversight | All in vivo experiments and protocols were approved by Institutional Animal Care and Use Committee of the Research Institute of Surgery, Daping Hospital IACUC protocol SCXK- (Army) 2007-017. |

Note that full information on the approval of the study protocol must also be provided in the manuscript.
