# Supplementary material for: Dstyk mutation leads to congenital scoliosis-like vertebral malformations in zebrafish via dysregulated mTORC1/TFEB pathway
Source: Nat Commun. 2020 Jan 24;11:479. doi: 10.1038/s41467-019-14169-z (PMC6981171; doi:10.1038/s41467-019-14169-z)
Supplement: Supplementary file 3 — Description of Additional Supplementary Files [file 41467_2019_14169_MOESM3_ESM.pdf]

Description of Additional Supplementary Files

Supplementary Movie 1. Live confocal time-lapse imaging of vacuole expansion for WT from 20 hpf to 30 hpf used internal membrane tracker. The time interval between frames is 10 min. Scale bar represent 20 μm.

Supplementary Movie 2. Live confocal time-lapse imaging of vacuole expansion for *dstyk* mutant from 20 hpf to 30 hpf used internal membrane tracker. The time interval between frames is 10 min. Scale bar represent 20 μm.
